# Proteome-Wide Analyses Provide New Insights into the Compatible Interaction of Rice with the Root-Knot Nematode *Meloidogyne graminicola*

**DOI:** 10.3390/ijms21165640

**Published:** 2020-08-06

**Authors:** Chao Xiang, Xiaoping Yang, Deliang Peng, Houxiang Kang, Maoyan Liu, Wei Li, Wenkun Huang, Shiming Liu

**Affiliations:** 1 State Key Laboratory for Biology of Plant Diseases and Insect Pests, Institute of Plant Protection, Chinese Academy of Agricultural Sciences, Beijing 100193, China; xiangchao2018@163.com (C.X.); pengdeliang@caas.cn (D.P.); Kanghouxiangcaas@163.com (H.K.); liu-mao-yan@foxmail.com (M.L.); 2 Hunan Biological and Electromechanical Polytechnic, Changsha 410127, China; xiaopingyang75@163.com; 3 College of Plant Protection, Hunan Agricultural University, Changsha 410128, China; liwei350551@163.com

**Keywords:** rice, root-knot nematode, *Meloidogyne graminicola*, compatible interaction, proteome-wide analyses

## Abstract

The root-knot nematode *Meloidogyne graminicola* is an important pathogen in rice, causing huge yield losses annually worldwide. Details of the interaction between rice and *M. graminicola* and the resistance genes in rice still remain unclear. In this study, proteome-wide analyses of the compatible interaction of the *japonica* rice cultivar “Nipponbare” (NPB) with *M. graminicola* were performed. In total, 6072 proteins were identified in NPB roots with and without infection of *M. graminicola* by label-free quantitative mass spectrometry. Of these, 513 specifically or significantly differentially expressed proteins were identified to be uniquely caused by nematode infection. Among these unique proteins, 99 proteins were enriched on seven Kyoto Encyclopedia of Genes and Genomes (KEGG) pathways. By comparison of protein expression and gene transcription, LOC_Os01g06600 (ACX, a glutaryl-CoA dehydrogenase), LOC_Os09g23560 (CAD, a cinnamyl-alcohol dehydrogenase), LOC_Os03g39850 (GST, a glutathione S-transferase) and LOC_Os11g11960 (RPM1, a disease resistance protein) on the alpha-linolenic acid metabolism, phenylpropanoid biosynthesis, glutathione metabolism and plant–pathogen interaction pathways, respectively, were all associated with disease defense and identified to be significantly down-regulated in the compatible interaction of NPB with nematodes, while the corresponding genes were remarkably up-regulated in the roots of a resistant rice accession “Khao Pahk Maw” with infection of nematodes. These four genes likely played important roles in the compatible interaction of rice with *M. graminicola*. Conversely, these disease defense-related genes were hypothesized to be likely involved in the resistance of resistant rice lines to this nematode. The proteome-wide analyses provided many new insights into the interaction of rice with *M. graminicola.*

## 1. Introduction

As one of the top ten plant-parasitic nematodes [1], the root-knot nematode (RKN) *Meloidogyne graminicola* is a tremendous threat to rice (*Oryza sativa* L.) worldwide. Recent cultivation pattern alterations and climate changes have further aggravated the damaging effect of this nematode [2,3,4]. This nematode mainly infects rice root tips and induces root cells to form giant cells as a nutrition resource with a characteristic symptom of hook-shaped galls on the roots, resulting in deficient growth and severe rice yield losses of up to 87% [5,6]. In well-drained soil, at 22–29 °C, the swelling of root tips can be vaguely observed at 1 day post inoculation (dpi), hook-shaped galls can be clearly visible on the root tips at 3 dpi, and at 7 to 15 dpi, following 1–2 times of molting, the nematodes eventually develop into females, which then lay eggs in the galls [7,8]. The short life cycle makes *M. graminicola* difficultly controlled [2]. Planting resistant rice cultivars is an eco-friendly and economical means to prevent and control this pathogen. Analyses of details of the interaction of rice with *M. graminicola* and identification of the *M. graminicola*-resistant proteins (genes) are of significance for the management of this nematode.

The resistance of rice to *M. graminicola* is controlled by quantitative trait loci (QTL). Galeng-Lawilao et al. (2018) [9] identified a total of 12 QTL underlying resistance and tolerance to *M. graminicola*. The identification of candidate genes underlying resistance to *M. graminicola* is mostly performed using transcriptomes obtained by RNA sequencing (RNA-seq). Kyndt et al. (2012) [10] identified 382 loci that were significantly differentially expressed in *M. graminicola*-infected galls following study of the transcriptional reprogramming patterns of galls induced in the compatible interaction between the *japonica* rice cultivar “Nipponbare”(NPB) and *M. graminicola* at 3 and 7 dpi using RNA-seq. Analyses of the transcriptome of NPB giant cells at 7 and 14 dpi by using both laser-capture microdissection and RNA-seq indicated that the expression of genes associated with chloroplast biogenesis and tetrapyrrole synthesis was notably changed, while the majority of defense-related genes were extremely suppressed by infection of *M. graminicola* [11]. Through comparison of the transcriptomes of rice accessions compatibly and incompatibly interacting with *M. graminicola* at 2, 4 and 8 dpi, 32 potential *M. graminicola*-resistance-associated genes such as phenylalanine ammonia lyase, thionin and chalcone synthase genes were identified [12]. However, the rice genes underlying resistance to *M. graminicola* are not yet clear.

Many genes (regulators) impact traits at translation level rather than at transcription level. For example, in the induction of immunity triggered by the microbe-associated molecular pattern elf18, translational efficiency of Arabidopsis was mediated by the R-motif in a highly enriched messenger RNA through interaction with poly(A)-binding proteins [13]. The proteome can provide accurate and direct bioinformation and may be better than the transcriptome for the identification of many genes (regulators) underlying traits. Recently, sole proteomics analyses or combination analyses with other omics have been applied to the interaction of rice with pathogens, such as the regulation by chitosan oligosaccharide of the expression of plant defense-related proteins and virus proteins in the interaction of rice with southern rice black-streaked dwarf virus [14], the interaction of rice with *Fusarium fujikuroi* [15], and the responses of diverse genotypes of rice to *Magnaporthe oryzae* [16]. In this study, on one hand, to gain new insights into the interaction of rice with the RKN *M. graminicola*, the susceptible rice cultivar NPB was selected to perform proteome-wide analyses of the compatible interaction with *M. graminicola* by label-free quantitative mass spectrometry. Through comparison between proteins with and without infection of nematodes, the specifically expressed proteins and the significantly differentially expressed proteins (SDEPs), and then the proteins uniquely caused by the infection of nematodes in the roots of NPB, were identified. On the other hand, because the *M. graminicola*-resistant genes are not yet identified in rice, we also tried to find some useful bioinformation on the resistance of rice against this nematode through the proteome-wide analyses. So far, most of the identified RKN-resistant genes such as *Mi-1*, *Mi-9* and *Ma* all belong to the nucleotide binding site-leucine-rich repeat (NBS-LRR) family [17,18,19,20,21]. The expression of this type of proteins was one of our primary targets to be studied. Rice genes/proteins on certain defense-related and plant–pathogen interaction pathways may be down-regulated in the compatible interaction of rice with nematodes that the defense of rice against nematodes is likely suppressed, resulting in susceptibility, while these identical genes/proteins are up-regulated in the incompatible interaction of rice with nematodes. Therefore, we paid more attention to this type of proteins and tried to identify proteins with opposite expression patterns in susceptible and resistant rice accessions using the unique proteins obtained by the proteome-wide analyses. For this, we confirmed a previously reported *M. graminicola*-resistant rice accession ‘‘Khao Pahk Maw’’ [22] and used it to analyze the transcriptional abundance of corresponding genes. Meanwhile, during the analyses, we noticed that the expressions of a vesicle-associated membrane protein (VAMP, LOC_Os10g06540), an aminotransferase domain-containing protein (AT, LOC_Os05g15530) and the PEX3 (Loc_Os09g14510) were down-regulated in the compatible interaction of NPB with nematodes. They are associated with SNARE interactions in vesicular transport, folate biosynthesis and peroxisome pathway, respectively. The vesicular transport-related GmSNAP18 and the folate biosynthesis-related GmSHMT08 have been cloned and functionally identified to play very important roles in the resistance of soybean to soybean cyst nematode, which is one of the devastating pathogens in soybean [23,24,25], and PEX3 is involved in the cleavage of hydrogen peroxide and ß-oxidation of very long chain fatty acids to short chain fatty acids, playing key roles in the conversion of bioactive oxygen species and lipid metabolism [26]. Therefore, these three proteins/genes were further analyzed in this study. According to the analysis results, besides VAMP, AT and PEX3, another five proteins, namely, LOC_Os01g06600 (ACX, a glutaryl-CoA dehydrogenase) on the alpha-linolenic acid metabolism pathway, LOC_Os09g23560 (CAD, a cinnamyl-alcohol dehydrogenase) on the phenylpropanoid biosynthesis pathway, LOC_Os03g39850 (GST, a glutathione S-transferase) on the glutathione metabolism pathway, and LOC_Os11g11960 (RPM1, a disease resistance protein) and LOC_Os02g46090 (CDPK, a calcium/calmodulin dependent protein kinase) on the plant–pathogen interaction pathway, were also studied in detail. The findings obtained in this study provide many new insights into the compatible interaction of rice with the RKN *M. graminicola*.

## 2. Results

### 2.1. Proteome Measurement of the Roots of Rice Cultivar NPB with or without Infection of M. graminicola

For the proteome measurement by label-free quantitative mass spectrometry (MS) technology, 14-day-old seedlings of the rice cultivar NPB were divided into two groups; one group (‘’MG’’) was inoculated and the other group (‘‘CK’’) was not inoculated with *M. graminicola*. The roots were collected at four time-points: before inoculation, and 1, 3 and 7 dpi of *M. graminicola*. The samples collected before inoculation were used as the control (CK0), and the other collected samples were correspondingly used as MG1DPI and CK1DPI (1 dpi), MG3DPI and CK3DPI (3 dpi), and MG7DPI and CK7DPI (7 dpi). Because the galls started to be obviously visible at 3 dpi [7,8], the root tips were collected at two time-points (before inoculation and 1 dpi), while the root parts mainly containing the galls were selected under stereomicroscopy and collected at the other two time-points (3 and 7 dpi). In this study, three biological replicates were set for each sample; each replicate was measured by label-free quantitative mass spectrometry independently, and their obtained proteome data were analyzed individually. A large number of proteins were quantitatively identified in each sample. In total, 6072 proteins were identified in the roots of NPB with and without infection of nematodes. Among them, 5821, 5862, 5860, 5773, 5822, 5844 and 5784 proteins were identified in CK0, CK1DPI, CK3DPI, CK7DPI, MG1DPI, MG3DPI and MG7DPI, respectively (Table 1). Compared to the total numbers of proteins in CK0, no significant difference was shown in the identified protein numbers among all the other measured samples.

The infection of nematodes causes not only the expression of new proteins but also changes in the expression levels of proteins (up- or down-regulated) in the host plants. The Volcano plot is a type of scatterplot that enables the identification of genes/proteins with large fold changes that are also of statistical significance. Because all the comparisons later on were carried out based on the specifically expressed proteins and the significantly differentially expressed proteins (SDEPs, up- and down-regulated) in the roots of each sample compared to CK0, we first conducted Volcano plot analyses to identify SDEPs in each sample using all the identified proteins. The results showed different patterns of SDEPs in the roots at various days with or without infection of *M. graminicola* as per the Volcano plots (Figure S1). Subsequently, to compare the similarity of proteome data among replicates, all the acquired SDEPs of each replicate were employed to perform hierarchical clustering analyses. The results showed that the three biological replicates in each sample had highly similar expression profiles (Figure S2), suggesting the reliability of the proteome data.

### 2.2. Proteome-wide Analyses of the Roots of NPB with Infection of M. graminicola

**Figure 1:**
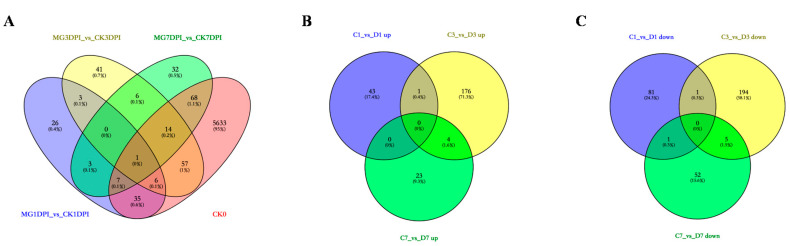
Proteomic profiles of roots of rice NPB with infection of *M. graminicola* during 7 days of growth: (**A**) specifically expressed proteins, (**B**) up-regulated significantly differentially expressed proteins (SDEPs), and (**C**) down-regulated SDEPs.

To gain insights into the compatible interaction of rice NPB with *M. graminicola*, proteome-wide comparisons were carried out between the roots of NPB with and without infection of *M. graminicola* (MG_versus_CK). Compared to the corresponding samples of the “CK” group (without nematode infection), the numbers of SDEPs, which are summarized from the details as shown in Figure S1A–C, and the specifically expressed proteins of the samples in the “MG” group (with nematode infection) are presented in Figure 1. The results showed that compared to the corresponding CK1DPI, CK3DPI and CK7DPI, in total, 111 proteins were newly emerged (Figure 1A), 247 SDEPs were up-regulated (Figure 1B) and 334 SDEPs were down-regulated (Figure 1C) among MG1DPI, MG3DPI and MG7DPI. Among these, 32, 50 and 41 specifically expressed proteins; 42, 181 and 27 up-regulated SDEPs; and 83, 200 and 58 down-regulated SDEPs were identified at 1, 3 and 7 dpi, respectively (Figure 1). Obviously, the numbers of both newly emerged specifically expressed proteins and SDEPs were higher at 3 dpi than at both 1 dpi and 7 dpi. In particular, the numbers of SDEPs at 3 dpi were much higher than those at both 1 and 7 dpi (Figure 1). In addition, the root materials used to perform MS measurement mainly contained the galls from 3 dpi onwards. All these results indicated that the gall cells might be most active at 3 dpi. Meanwhile, these results also indicated that the infection of nematodes might considerably induce or suppress the expression of proteins in the roots of NPB.

**Figure 2:**
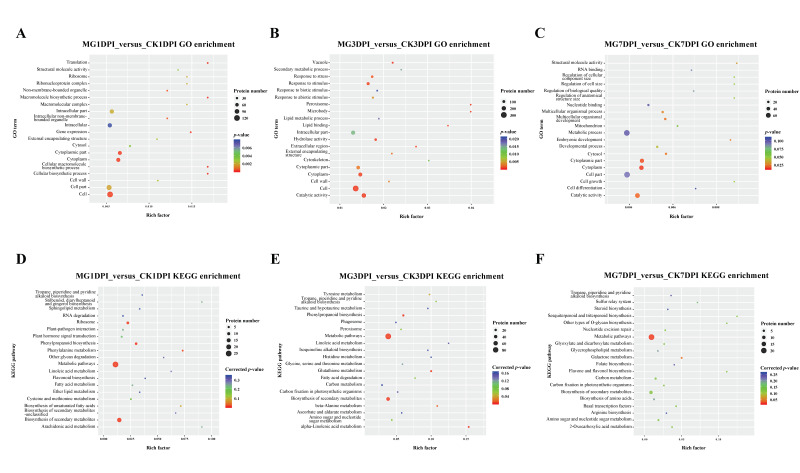
GO and KEGG enrichment analyses using all the specifically expressed proteins and SDEPs of NPB roots in MG1DPI_versus_CK1DPI, MG3DPI_versus_CK3DPI, or MG7DPI_versus_CK7DPI. (**A**–**C**) GO enrichment analyses in MG1DPI_versus_CK1DPI, MG3DPI_versus_CK3DPI, and MG7DPI_versus_CK7DPI, respectively. (**D**–**F**) KEGG enrichment analyses in MG1DPI_versus_CK1DPI, MG3DPI_versus_CK3DPI, and MG7DPI_versus_CK7DPI, respectively.

Successively, using all the specifically expressed proteins and SDEPs from MG1DPI_versus_CK1DPI, MG3DPI_versus_CK3DPI or MG7DPI_versus_CK7DPI, the enrichment of Gene Ontology (GO) and Kyoto Encyclopedia of Genes and Genomes (KEGG) pathways was analyzed separately. The results (only the 20 most significantly enriched GO terms and KEGG pathways in each comparison are shown, Figure 2) showed that the significance and quantity of proteins enriched in GO and KEGG terms were different at various dpi. In particular, the proteins in the terms “vacuole”, “response to stress”, “response to stimulus” and “peroxisome” were significantly enriched at 3 dpi (Figure 2B), while no proteins in these terms were enriched at 7 dpi (Figure 2C). These terms are all relevant to the defense of plants against stress including pathogens. These results indicated that the host rice most strongly responded to the infection of nematodes at 3 dpi. Regarding the KEGG enrichment analyses, the proteins in the terms “metabolic pathways” and “biosynthesis of secondary metabolites” were significantly enriched at both 1 and 3 dpi. However, at 7 dpi, the proteins were not significantly enriched in the term “biosynthesis of secondary metabolites”, despite the proteins being significantly enriched in “metabolic pathways” (Figure 2D–F).

**Figure 3:**
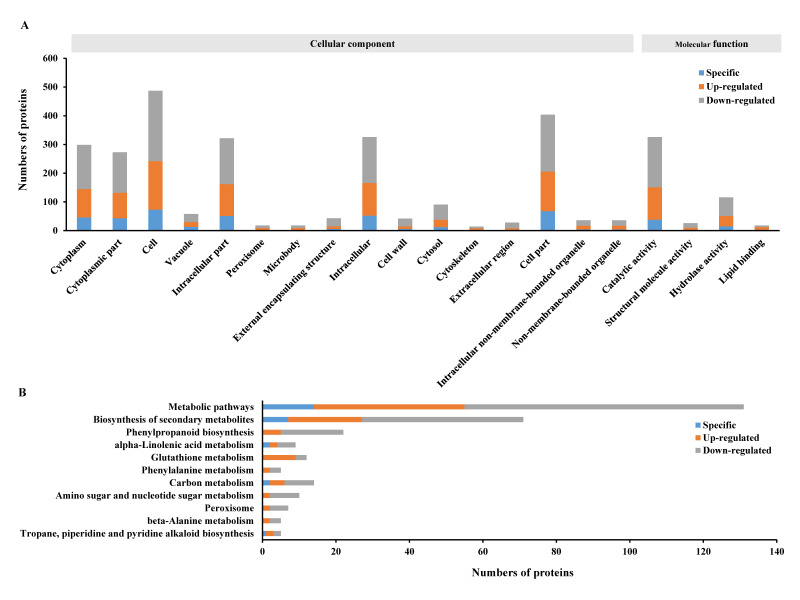
GO and KEGG enrichment analyses using all the specifically expressed proteins and SDEPs of NPB roots in the whole MG_versus_CK group: (**A**) GO enrichment analyses, and (**B**) KEGG pathway enrichment analyses.

Subsequently, all the specifically expressed proteins and SDEPs in the entire MG_versus_CK group were put together to perform GO and KEGG enrichment analyses (Figure 3). The results revealed that 289, 263 and 460 proteins associated with the “cytoplasm”, “cytoplasmic part” and “cell” GO terms (Figure 3A), and 123, 67 and 19 proteins involved in “metabolic pathways”, “biosynthesis of secondary metabolites” and “phenylpropanoid biosynthesis” out of the 11 KEGG pathways (Figure 3B), respectively, were most significantly enriched. These results suggested that the infection of *M. graminicola* mainly induced significant changes in cellular components as well as molecular functions including many types of metabolic biosynthesis pathways in the host rice NPB.

### 2.3. Proteins in the Roots of NPB Uniquely Caused by Infection of M. graminicola

**Figure 4:**
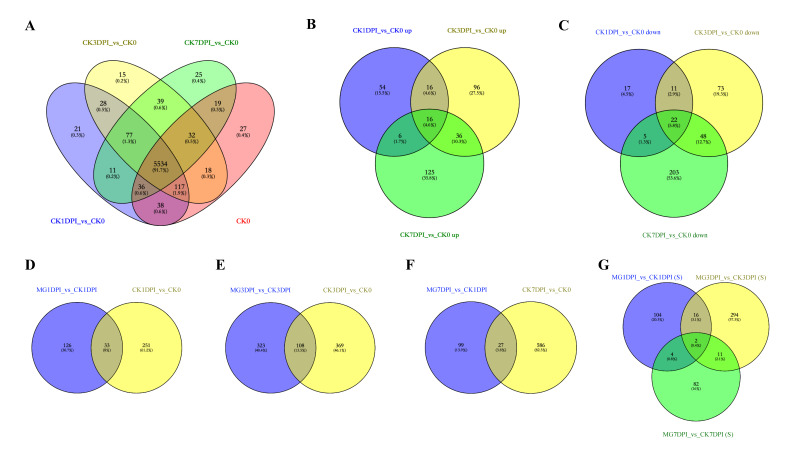
Proteins in the roots of NPB uniquely caused by infection of *M. graminicola*. (**A**–**C**) Specifically expressed proteins, up-regulated significantly differentially expressed proteins (SDEPs) and down-regulated SDEPs in the roots of NPB without infection of *M. graminicola* at 1, 3 and 7 days of growth, respectively (CK_versus_CK0). (**D**–**F**) Comparison of the proteins (all the specifically expressed proteins and up- and down-regulated SDEPs) in the roots of NPB between samples with and without infection of *M. graminicola* at 1, 3 and 7 dpi, respectively (MG_versus_CK and CK_versus_CK0). (**G**) All the unique proteins in the roots of NPB with infection of *M. graminicola* at 1, 3 and 7 dpi (the blue parts in (**D**–**F**)).

Firstly, compared to the proteins in CK0, the specifically expressed proteins and SDEPs were analyzed using the data of samples in the “CK” group (CK1DPI, CK3DPI and CK7DPI) to show the proteome changes of the NPB seedling roots without infection of *M. graminicola* (CK_versus_CK0). The numbers of specifically expressed proteins are shown in Figure 4A, and the numbers of SDEPs summarized from Figure S1D–F are exhibited in Figure 4B,C. In total, 216 proteins were specifically expressed in the “CK” group, including 137 proteins in CK1DPI, 159 proteins in CK3DPI and 152 proteins in CK7DPI, with 77 proteins shared among CK1DPI, CK3DPI and CK7DPI (Figure 4A). A total of 349 SDEPs were up-regulated including 92 SDEPs in CK1DPI, 164 SDEPs in CK3DPI and 183 SDEPs in CK7DPI, with 16 SDEPs shared among CK1DPI, CK3DPI and CK7DPI (Figure 4B). A total of 379 SDEPs were down-regulated including 55 SDEPs in CK1DPI, 154 SDEPs in CK3DPI and 278 SDEPs in CK7DPI, with 22 SDEPs shared among CK1DPI, CK3DPI and CK7DPI (Figure 4C).

Secondly, all the obtained specifically expressed proteins and SDEPs in the “CK” group compared to CK0 were also employed to perform GO and KEGG enrichment analyses. The detailed results of GO and KEGG enrichment analyses in CK1DPI_versus_CK0, CK3DPI_versus_CK0 or CK7DPI_versus_CK0 are shown in Figure S3 (just the 20 most significantly enriched terms are shown), while the summarized enrichment results using all the specifically expressed proteins and SDEPs in the whole CK_versus_CK0 group are displayed in Figure S4. The GO enrichment analyses indicated that many proteins associated with “biological process”, “cellular component” and “molecular function” were significantly enriched. In particular, 63, 64 and 69 proteins involved in “ribosome”, “structural molecule activity” and “cellular biosynthetic process”, respectively, were most significantly enriched (Figure S4A). The KEGG enrichment analyses indicated that of the 25 significantly enriched KEGG pathways, 180, 55 and 85 proteins involved in “metabolic pathways”, “ribosome” and “biosynthesis of secondary metabolites”, respectively, were most significantly enriched (Figure S4B). All these results indicated that the expressed proteins in NPB seedling roots without infection of *M. graminicola* were being dynamically as well as dramatically changed along with the 7 days of growth at this seedling stage.

Thirdly, we compared the proteins between MG_versus_CK (Figure 1) and CK_versus_CK0 (Figure 4A–C). There were some common proteins shared between the two groups at each time-point: 33, 108 and 27 proteins at 1, 3 and 7 dpi, respectively (Figure 4D–F). These results suggested that infection of nematodes not only caused dramatic changes in feeding site giant cells (galls) but also might impact the growth of rice roots and even of the whole seedlings. To concentrate on the changes in the galls at protein level and to gain insights into the accurate compatible interaction of rice with nematodes, we mainly focused on the proteins after removal of the common proteins shared between MG_versus_CK and CK_versus_CK0 at each time-point. Of the 656 proteins in the MG_versus_CK group, 513 unique proteins were obtained (Figure 4G), while 143 proteins were shared with the CK_versus_CK0 group. The detailed unique protein information is listed in Table S1. Obviously, these proteins in the roots of NPB seem to be uniquely caused by the infection of *M. graminicola*.

### 2.4. Important Proteins in the Roots Associated with the Compatible Interaction of NPB with M. graminicola

**Figure 5:**
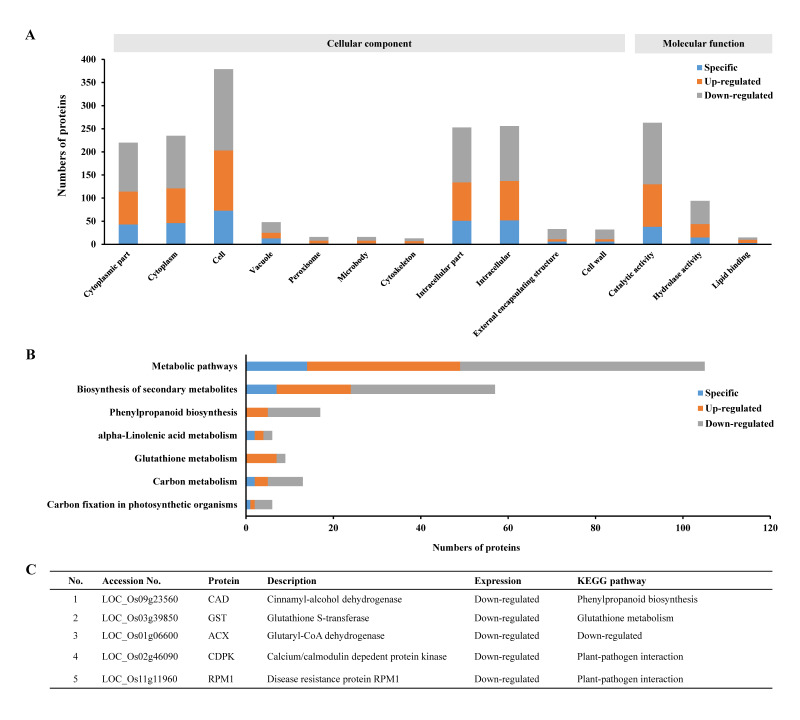
Proteins in the roots associated with the interaction of NPB with *M. graminicola*: (**A**) GO enrichment analyses using all the unique proteins in Figure 4G, (**B**) KEGG pathway enrichment analyses using all the unique proteins in Figure 4G, and (**C**) information on the selected proteins in the roots associated with the interaction of rice with *M. graminicola*.

All the 513 unique proteins identified above were then used in GO and KEGG enrichment analyses. The GO enrichment analyses showed that 402 proteins were involved in 14 significantly enriched GO terms related to cellular components and molecular functions. Of these, the proteins associated with “cytoplasmic part”, “cytoplasm” and “catalytic activity” were most significantly enriched (Figure 5A). The KEGG enrichment analyses indicated that only 99 proteins involved in 7 pathways (there were 62 proteins involved in 2–4 pathways, Table S1) were significantly enriched (Figure 5B). Obviously, the numbers of enriched proteins and the quantity of enrichment GO terms and KEGG pathways (Figure 5A,B) were both less than those without removal of the common proteins shared with the CK_versus_CK0 group (Figure 3), simplifying the subsequent analyses.

Of the 7 KEGG pathways (Figure 5B), 3 pathways, namely, phenylpropanoid biosynthesis, glutathione metabolism and alpha-linolenic acid metabolism, have been reported to be involved in the defense against stresses. There were 28 enriched proteins on these 3 pathways: 14 proteins on phenylpropanoid biosynthesis, 8 proteins on glutathione metabolism and 6 proteins on alpha-linolenic acid metabolism (Figure 5B). Of these, 3 proteins were down-regulated, namely, LOC_Os09g23560 (CAD, a cinnamyl-alcohol dehydrogenase) on the phenylpropanoid biosynthesis pathway, LOC_Os03g39850 (GST, a glutathione S-transferase) on the glutathione metabolism pathway and LOC_Os01g06600 (ACX, a glutaryl-CoA dehydrogenase) on the alpha-linolenic acid metabolism pathway (Figure 5C, Table S1). The down-regulation of the defense genes on these 3 pathways might contribute to the compatible interaction of NPB with *M. graminicola*. In addition, there were 2 down-regulated proteins, LOC_Os02g46090 (CDPK, a calcium/calmodulin dependent protein kinase) and LOC_Os11g11960 (RPM1, a disease resistance protein), on the plant–pathogen interaction pathway in the compatible interaction of NPB with *M. graminicola* (Figure 5C, Table S1). These 2 proteins might also be involved in the compatible interaction. Thus, these 5 proteins were identified as the important proteins in the roots associated with the compatible interaction of NPB with *M. graminicola*.

### 2.5. Proteins with Opposite Expression Patterns in Susceptible and Resistant Rice Lines in Response to the Infection of M. graminicola

**Figure 6:**
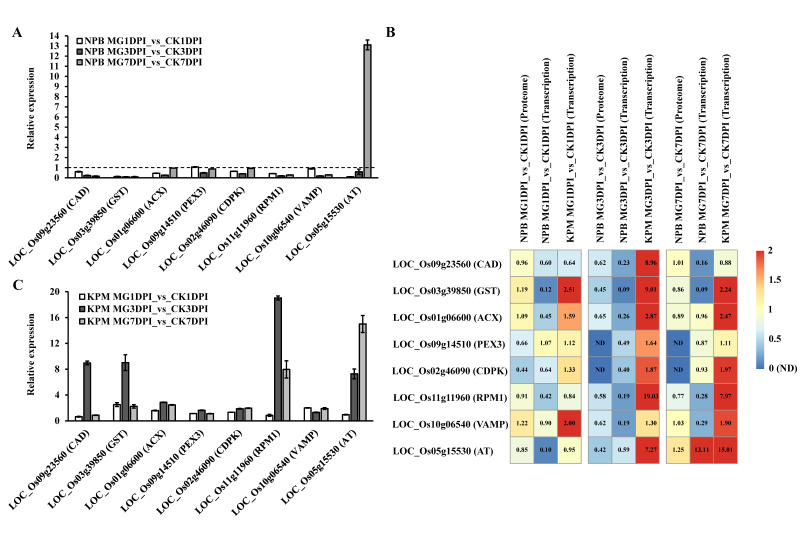
Identification of proteins with opposite expression patterns in resistant and susceptible rice lines in response to the infection of *M. graminicola*. (**A**) Transcriptional abundance of genes encoding 8 selected proteins in the roots of NPB before and 1, 3 and 7 dpi of *M. graminicola*. (**B**) Detailed data of proteome and transcription of the 8 selected proteins/genes in the roots of NPB and Khao Pahk Maw. KPM, Khao Pahk Maw; ND, not detectable. All the numbers denote fold changes compared to CK0. (**C**) Transcriptional abundance of genes encoding 8 selected proteins in the roots of the resistant rice accession Khao Pahk Maw before and 1, 3 and 7 dpi of *M. graminicola*. KPM, Khao Pahk Maw.

We first analyzed the reliability of the proteome data by comparison of the expression trends of proteins and transcripts of the corresponding genes. Besides the five above-mentioned proteins’ genes, three genes, namely, *VAMP* (*LOC_Os10g06540*) on the SNARE interactions in vesicular transport pathway, *AT* (*LOC_Os05g15530*) on the folate biosynthesis pathway and *PEX3* (*LOC_Os09g14510*) on the peroxisome pathway, were also selected to analyze their transcriptional abundance in the roots of rice before and after infection of *M. graminicola*. As per the proteome data, all the eight selected proteins were down-regulated in *M. graminicola*-infected NPB at one to three time-points (1, 3 and 7 dpi) (Figure 5C, Table S1). The transcriptional abundance results indicated that *AT* was significantly up-regulated in NPB at 7 dpi (Figure 6A), and its encoded protein was slightly up-regulated (0.25 fold changes, Figure 6B) at 7 dpi. In contrast, all the other seven genes were down-regulated in the NPB roots at most of or all of the time-points (Figure 6A), almost matching the trends of expression of the corresponding proteins (Figure 6B). These results suggested that the obtained quantitative proteome data were reliable.

Subsequently, we confirmed one *M. graminicola*- highly resistant rice accession, Khao Pahk Maw, which was identified to be resistant to *M. graminicola* [22]. In the scoring at 14 dpi, only one gall was observed on 189 roots of Khao Pahk Maw with the gall index (GI) of 1.1, while the GI of the susceptible cultivar NPB was 52.1.

The Khao Pahk Maw accession was then used to analyze the transcriptional abundance of the eight selected genes in the roots before and after inoculation at different time-points. The results showed that among the eight selected genes, the transcriptional abundance of *ACX*, *CAD*, *GST*, *RPM1* and *AT* was all significantly up-regulated (relative expression value > 2), while the transcription abundance of *PEX3*, *CDPK* and *VAMP* was not remarkably changed (relative expression value ≤ 2), in the nematode-infected roots compared to the non-infected roots of Khao Pahk Maw (Figure 6C). Comparing the transcription and proteomics data of the roots in susceptible NPB and resistant Khao Pahk Maw for the eight proteins/genes, clearly only ACX, CAD, GST and RPM1 showed opposite reactions in the susceptible and resistant rice lines that all of them were significantly down-regulated in susceptible NPB, whereas they were remarkably up-regulated in resistant Khao Pahk Maw, in response to the infection of *M. graminicola* (Figure 6A–C).

## 3. Discussion

The interaction of plants with plant-parasitic root-knot nematodes is usually studied by transcriptomic and/or histocytological analyses, but proteome-wide analyses have also been applied in this field and have provided promising results. Recently, combination analyses of quantitative proteomics with RNA-seq on a resistant cucumber cultivar “IL10-1” and a susceptible cucumber cultivar “CC3” revealed the important roles of the MAPK signaling and flavonoid metabolic pathway in the resistance of cucumber to *M. incognita* [27]. In this study, we conducted proteome-wide analyses of the compatible interaction of NPB rice with *M. graminicola* (Figure S5A,B). A large number of proteins were quantitatively identified in each sample by label-free quantitative MS measurement (Table 1). There were more newly emerged proteins and up- and down-regulated SDEPs in the roots of NPB at 3 dpi than at both 1 and 7 dpi (Figure 1). In addition, the galls started to be noticeably visible at 3 dpi [7,8]. Therefore, it may be concluded that the feeding site giant cells in roots of rice were most active at 3 dpi of nematodes, they needed to biosynthesize much more substances for the growth of feeding site giant cells (galls) in the interaction. Further, compared to before inoculation (CK0), at 3 dpi, there were 181 up-regulated SDEPs and 200 down-regulated SDEPs, the quantity of SDEPs was much more than that of the newly emerged proteins (50) in the infected roots (Figure 1). Therefore, the infection of nematodes might largely impact (stimulate or suppress) the expression of proteins in the rice roots. Previously, transcriptomic analyses of NPB giant cells indicated that the majority of defense-related genes were enormously suppressed after infection of *M. graminicola* [11]. In this study, many proteins were also significantly enriched in several defense-related GO terms such as “vacuole”, “response to stress”, “response to stimulus” and “peroxisome” at 3 dpi, while no proteins were enriched in these terms at both 1 and 7 dpi (Figure 2A–C). Moreover, the proteins were significantly enriched on the KEGG pathway “biosynthesis of secondary metabolites” at 1 and 3 dpi rather than at 7 dpi (Figure 2D–F). Taken together, these results suggest that the rice roots were likely to still be strongly establishing the compatible interaction with nematodes before and at 3 dpi, until 7 dpi such an interaction might already be completely stable.

While comparing the proteome data between MG_versus_CK (Figure 1) and CK_versus_CK0 (Figure 4A–C) at identical time points, there were still a partiality of common proteins at each time-point (Figure 4D–F). In total, 143 proteins were shared between these two groups, a decrease of around 21.8% in quantity relative to the whole protein numbers (656) in these groups, suggesting that the changes in these common proteins might not be directly related to the compatible interaction of rice with *M. graminicola*. Of the 513 uniquely changed proteins (Figure 4G, detailed in Table S1), 402 and 99 proteins were enriched by GO and KEGG analyses, respectively (Figure 5A,B). These proteins are involved in the biosynthesis of many cellular components and are associated with many pathways, with the largest numbers of proteins in “cell” and “catalytic activity” in the GO terms and “metabolic pathways” and “biosynthesis of secondary metabolites” on the KEGG pathways (Figure 5A,B). All these results suggest that complicated but dramatic changes in cells took place in the compatible interaction of rice with *M. graminicola*. The changes in the abundancy of the uniquely enriched proteins are therefore identified as being likely associated with the susceptibility of NPB to *M. graminicola*.

*PEX3* on the peroxisome pathway for cleavage of hydrogen peroxide and ß-oxidation of very long chain fatty acids to short chain fatty acids was reported to be remarkably up-regulated by 4.13 fold changes in NPB at 7 dpi of *M. graminicola* by RNA-seq [11,26]. In contrast, in this study, the protein PEX3 was down-regulated at 1 dpi but not detectable at 3 and 7 dpi (Figure 6B), while the transcriptional abundance of *PEX3* was significantly decreased in NPB at 3 dpi but not significantly changed at 1 and 7 dpi (Figure 6A,B). The expression of VAMP involved in SNARE interactions in vesicular transport was not significantly changed in proteome data, whereas it was considerably decreased in transcription data at 7 dpi (Figure 6B). The expression of AT involved in folate biosynthesis was not changed much at 1 dpi but was slightly increased at 7 dpi in proteome data, while it was remarkably reduced at 1 dpi and significantly increased at 7 dpi in transcription data (Figure 6B). All these results obtained from this study and RNA-seq analyses by Ji et al. (2013) [11] obviously suggest different patterns of the same proteins/genes between protein and gene transcription expression in some cases. Multiple omics analyses will be much better to dissect the mechanism of compatible interaction of rice with nematodes. However, the substantial bioinformation acquired by proteome-wide analyses in this study provides many new insights into the compatible interaction of rice with *M. graminicola*.

So far, sources of *M. graminicola*-resistant rice are very limited [2,22,28,29] and the *M. graminicola*-resistant genes are not yet clear in rice, despite the fact that some resistance candidate genes were identified mainly through analyses of the transcriptomes by RNA-seq [10,11,12]. In this study, we confirmed the high resistance of the rice accession Khao Pahk Maw [22]. To conduct the proteome measurement for the interaction analyses, we chose to perform proteome-wide analyses of the compatible interaction of NPB with *M. graminicola*. Because the previously identified RKN-resistant genes *Mi-1*, *Mi-9* and *Ma* all belong to the NBS-LRR gene family [17,18,19,20,21], in this study, we first searched for significantly differentially expressed NBS-LRR proteins. However, no NBS-LRR proteins were enriched in the compatible interaction by proteome-wide analyses (Table S1). Analyses of the seven pathways carrying the 99 uniquely enriched proteins (Figure 5B, Table S1) indicated that three pathways, namely, alpha-linolenic acid metabolism, glutathione metabolism and phenylpropanoid biosynthesis, were noticeably reported to be involved in the defense of the plant against stresses including pathogens. For example, a glutathione S-transferase Fhb7 on the glutathione metabolism pathway was recently identified to confer resistance to Fusarium head blight in wheat [30]. Thus, these three pathways were most likely associated with the compatible interaction of NPB with nematodes. Of the 28 proteins associated with these three pathways, an ACX on the alpha-linolenic acid metabolism pathway, a CAD on the phenylpropanoid biosynthesis pathway and a GST on the glutathione metabolism pathway were all down-regulated during the compatible interaction with *M. graminicola* (Figure 5C, Table S1). In addition, an RPM1 and CDPK on the plant–pathogen interaction pathway were also down-regulated in the roots of NPB by infection of *M. graminicola* (Table S1). Among the genes encoding these five important proteins, four genes, *ACX*, *CAD*, *GST* and *RPM1*, were significantly up-regulated in the *M. graminicola*-highly resistant rice accession Khao Pahk Maw (Figure 6C) after infection of nematodes (Figure 6B,C), in contrast to the expression in the roots of susceptible rice NPB under infection conditions (Figure 6A,B). The down-regulation of these four genes might suppress the defense of rice against nematodes, so they likely played important roles in the compatible interaction of rice with *M. graminicola*. Conversely, these four genes are all associated with disease defense and are therefore hypothesized to be likely involved in the resistance of resistant rice lines to *M. graminicola*, all of which are different from the candidates previously reported to be involved in the resistance of rice to *M. graminicola*, to the best of our knowledge. However, due to no significant increase being found in the expression of *CDPK*, which is a calcium/calmodulin dependent protein kinase responsible for propagating immune signaling for the resistance of plants to pathogens [31], in resistant Khao Pahk Maw (Figure 6C), it may not be involved in the interaction of rice with *M. graminicola*. Regarding these four identified genes, the transcription expression of *RPM1* has been recorded by RNA-seq data, whereas RNA-seq data were not available for the other three genes [11]. The expression results of *RPM1* obtained in this study (Figure 6A,B) are consistent with the expression trend in RNA-seq data showing that *RPM1* was significantly down-regulated by 6.0 fold changes in NPB at 7 dpi of *M. graminicola* [11]. *RPM1* was identified as a resistant gene against the bacterium *Pseudomonas syringae* many years ago [32]. We strongly hypothesize that *RPM1* may confer resistance to *M. graminicola* in rice, and that it may possess broad-spectrum resistance against pathogens including the parasitic nematode *M. graminicola*. However, this needs to be extensively investigated.

In addition, there were 12 specifically expressed proteins, many up-regulated proteins and some other down-regulated proteins in NPB within the 99 enriched proteins identified on the seven KEGG pathways (Table S1). Of the 513 proteins uniquely caused by the infection of nematodes (Table S1), 402 proteins were enriched in 14 GO terms associated with cellular components and molecular functions (Figure 5A), some of which overlapped with the 99 proteins on the seven identified KEGG pathways (Table S1). These proteins may also be involved in the compatible interaction of NPB with nematodes. More detailed study of the compatible interaction of rice with *M. graminicola* is recommended in combination with other omics analyses on the basis of the proteomic data acquired in this study.

## 4. Materials and Methods

### 4.1. Rice Planting, Nematode Inoculation and Resistance Evaluation

The rice cultivar NPB and the *O. sativa* L. subsp. *Aus* accession Khao Pahk Maw that originated from Japan and Thailand and were reported to be susceptible and resistant to *M. graminicola*, respectively [11,22], were used as the experimental materials in this study. Their seeds were obtained from the International Rice Germplasm Center. The rice seeds germinated and were transplanted as described by Zhan et al. (2018) [33] with slight modifications. Briefly, the rice seeds were first soaked in 5.25% sodium hypochlorite (NaClO) for 10 min and germinated in the dark for 6 d at 28 °C; afterwards, each germinated seed was transferred into a polyvinylchloride tube containing SAP (sand and water-absorbent synthetic polymer) [34], growing under a 16 h/8 h light/dark regime at 75% relative humidity at 26 °C. Extra care was taken to irrigate each tube with 20 mL of Hoagland’s solution every three days.

*M. graminicola* was propagated and extracted as described by Huang et al. (2015) [35]. Fourteen-day-old plants were inoculated with 300 stage juveniles (J2s) of *M. graminicola* per plant. The control plants were inoculated with water. Resistance/susceptibility scoring was conducted using the methods described by Zhan et al. (2018) [36]. The gall index (GI) at 14 dpi was used to score resistance/sensitivity. The evaluation criteria were as follows: immune (I) GI = 0; highly resistant (HR) 0.1 ≤ GI ≤ 5.0; resistant (R) 5.1 ≤ GI ≤ 25.0; moderately susceptible (MS) 25.1 ≤ GI ≤ 50.0; susceptible (S) 50.1 ≤ GI ≤ 75.0; highly susceptible (HS) GI > 75.0.

Before the protein MS measurement, we observed the phenotype of the NPB with the infection of *M. graminicola*. After inoculation of *M. graminicola* on the roots of rice cultivar NPB, galls started to be clearly visible on the roots at 3 dpi [7,8]; many galls emerged on the roots at 7 dpi (Figure S5A). The galls were stained using the method described by Bybd et al. (1983) [37] with minor modifications. Briefly, the roots of the seedlings were successively treated with 5% NaClO for 10 m, rinsed thoroughly with water, and boiled in an acid fuchsin solution (5% acid fuchsin in 25% acetic acid) for 1 m. Subsequently, the seedlings were transferred to glycerin for decolorization. After staining, the nematodes inside the roots were observed under a stereoscope (Leica S9E, Leica, Wetzlar, Germany). Several nematodes were observed in each gall at 7 dpi (Figure S5B). These results indicated that NPB showed compatible interaction with *M. graminicola*, consistent with the results reported previously [11].

### 4.2. Sample Collection

For both NPB and Khao Pahk Maw, three samples were collected before inoculation as the controls (CK0). Additionally, regarding NPB, the samples of *M. graminicola*-infected root tips or the root parts mainly containing visible galls were collected from 3 seedlings at 1, 3 and 7 dpi. About 10–15 rice galls were mixed as one sample up to 500 mg at each time-point. All the samples were immediately frozen in liquid nitrogen and stored at −80 °C for further usage. The NPB samples were used for both proteome measurement and quantitative real-time PCR (qRT-PCR) analyses, while the samples of Khao Pahk Maw were used for qRT-PCR analyses only. Three biological replicates were set for each control or treatment, and each replicate was used as an independent sample.

### 4.3. Protein Extraction and Digestion

The proteins were extracted as described previously with slightly modifications [38]. Briefly, powder ground in liquid nitrogen was suspended in precooled trichloroacetic acid/acetone (*v:v* = 1:9) overnight and then centrifuged at 14,000× *g* for 15 min, and the precipitate was washed thrice with acetone following each time of centrifuge. White powder was re-suspended in SDT lysis buffer (4% SDS, 100 mM Tris-HCl, 1 mM DTT, 1 mM PMSF, pH7.6, including one-fold PhosSTOP phosphatase inhibitor mixture from Roche). The protein digestion was primarily performed as per the Filter Aided Sample Preparation (FASP) protocol [39]. Protein concentration was determined using the bicinchoninic acid (BCA) method [40].

### 4.4. High Performance Liquid Chromatography–Tandem Mass Spectrometry (HPLC–MS/MS)

Digested peptide mixtures were pressure-loaded onto a fused silica capillary column packed with 3 μm dionex C18 material (Phenomenex, Tianjin, China). The RP (reverse phase) sections with 100Å were 15 cm long, and the column was washed with buffer A (water containing 0.1% formic acid) and buffer B (acetonitrile containing 0.1% formic acid). After desalting, a 5 mm, 300 μm C18 capture tip was placed in line with an Agilent 1100 quaternary HPLC (Agilent, California, USA), and the samples were analyzed using a 12-step separation.

The first step consisted of a 5-min gradient from 0% to 2% buffer B, followed by a 45-min gradient to 40% buffer B, and then by gradient from 40% to 80% buffer B for 3 min and 10 min of 80% buffer B. After a 2-min buffer B gradient from 80% to 2%, approximately 100 μg of tryptic peptide mixture was loaded onto the columns and was separated at a flow rate of 0.5 μL/min by using a linear gradient. As peptides were eluted from the micro-capillary column, they were electrosprayed directly into a Bruker micrOTOF-Q II mass spectrometer (Bruker Daltonics, Billerica, MA, USA) with the application of a distal 180 °C source temperature. The mass spectrometer was operated in the MS/MS (auto) mode. Survey MS scans were acquired in the TOF-Q II with the resolution set to a value of 20,000. Each survey scan (50~2500) was followed by five data-dependent MS/MS scans at 2 Hz normalized scan speed.

### 4.5. Sequence Database Search and Data Analyses

Proteome Discoverer 2.1 (Thermo Fisher Scientific, Waltham, MA, USA) software was used for data analyses. Peptide identification was performed with the SEQUST search engine using the NPB proteome databases downloaded from the Rice Genome Annotation Project (http://rice.plantbiology.msu.edu/index.shtml) (access on 1 January 2019). Decoys for the database search were generated with the revert function. The following options were used to identify the proteins: Peptide mass tolerance = ±10 ppm, MS/MS tolerance = 0.02 Da, enzyme = trypsin, max missed cleavage = 2, fixed modification: Carbamidomethyl (C), variable modification: oxidation (M) and Acetyl (Protein N-term), database pattern = decoy. The false discovery rate (FDR) for peptides and proteins was set to 0.01.

The proteins only identified in one biological replicate were ignored (the same below), and only the up- or down-regulated proteins in at least 2 replicates with relative quantification *p*-value < 0.05 and 1.5 fold changes were selected as the SDEPs in the data using Cuffdiff [41].

### 4.6. Volcano Plot and Hierarchical Clustering Analyses

The Volcano plot and hierarchical clustering analyses were performed in the R environment (https://www.R-project.org) (access on 1 July 2020). The aes function in the package ggplot2 was used for Volcano plot drawing (https://CRAN.R-project.org/package=ggplot2) (access on 1 July 2020). The scale and hclust functions in the package stats were used for z-score normalization and hierarchical clustering, and the pheatmap function in the package pheatmap was employed for hierarchical clustering drawing (https://CRAN.R-project.org/package=pheatmap) (access on 1 July 2020).

### 4.7. Bioinformatic Analysis

The agriGO v2.0 [42] software was employed for Gene Ontology (GO) annotation and enrichment analyses, and the Kyoto Encyclopedia of Genes and Genomes (KEGG) was used for pathway annotation and enrichment analyses [43] to gain a better understanding of the functions of the selected proteins. The GO enrichment on three ontologies, including biological process, molecular function and cellular component, and the KEGG pathway enrichment were applied on the basis of Fisher’s exact test, and only the GO categories and KEGG pathways with *p*-value < 0.05 and FDR < 0.05 or corrected *p*-value < 0.05 were considered as significant enrichment.

### 4.8. RNA Extraction and qRT-PCR

Total RNA was extracted from galls/roots using the RNeasy Plant Mini Kit (Qiagen, Dusseldorf, Germany), and cDNA was synthesized using the PrimeScript™ RT reagent Kit with gDNA Eraser (Perfect Real Time) (TaKaRa, Tokyo, Japan) as per the manufacturer’s instructions. The quality and concentration of extracted RNA were measured using a Nanodrop 2000 (Thermo Scientific, Waltham, MA, USA).

The expression of the 8 selected genes in NPB and Khao Pahk Maw was measured by qRT-PCR using the designed primers listed in Table S2. The qRT-PCR was performed on an ABI 7500 Fast Real Time PCR System (Thermo Fisher Scientific, Waltham, MA, USA). The ubiquitin gene *UBQ5* (*LOC_Os01g22490*) was used as the reference gene [44]. Three replicates were set for each sample. Relative gene expression level was calculated using the 2^−ΔΔCt^ method [45].

### 4.9. Statistical Analyses of Data

The statistical significance of all the data was analyzed by ANOVA using SPSS version 25 (IBM, Armonk, NY, USA). All the data were analyzed using a paired *t*-test and Duncan’s multiple comparison test.

## Figures and Tables

**Table 1:** General information of proteome measurement of the seedling roots of NPB by label-free quantitative mass spectrometry. The numbers represent the numbers of proteins quantitatively identified. CK0; MG1DPI and CK1DPI; MG3DPI and CK3DPI; and MG7DPI and CK7DPI denote the root samples just before infection and at 1, 3 and 7 dpi with (‘MG’) and without (‘CK’) infection of *M. graminicola*, respectively.

Samples	Repeat 1	Repeat 2	Repeat 3	Total	Common in All 3 Repeats	Common Only in 2 Repeats
CK0	5801	5819	5683	5821	5473	348
CK1DPI	5784	5838	5824	5862	5568	294
CK3DPI	5861	5774	5771	5860	5503	357
CK7DPI	5702	5763	5692	5773	5379	394
MG1DPI	5795	5760	5797	5822	5538	284
MG3DPI	5821	5769	5772	5844	5511	333
MG7DPI	5734	5757	5744	5784	5482	302
Average				5824		
Total				6072		

## Supplementary Materials

Supplementary materials can be found at https://www.mdpi.com/1422-0067/21/16/5640/s1. Figure S1: Volcano plots of the relative protein abundance changes of all the identified proteins in the roots of NPB with or without infection of *M. graminicola*. Figure S2: Clustering heatmaps of the three biological replicates. Figure S3: GO and KEGG enrichment analyses using all the specifically expressed proteins and SDEPs of NPB roots in CK1DPI_versus_CK0, CK3DPI_versus_CK0 or CK7DPI_versus_CK. Figure S4: GO and KEGG enrichment analyses of the specifically expressed proteins and SDEPs of the NPB roots in the whole CK_versus_CK0 group. Figure S5: Compatible interaction of the *japonica* rice cultivar NPB with *M. graminicola*. Table S1: List of the specifically expressed proteins and significantly differentially expressed proteins (SDEPs) in the roots of NPB rice uniquely caused by the infection of *M. graminicola*. Table S2: List of the primers used in this study.
