# Supplementary material for: Proteome-Wide Analyses Provide New Insights into the Compatible Interaction of Rice with the Root-Knot Nematode Meloidogyne graminicola
Source: Int J Mol Sci. 2020 Aug 6;21(16):5640. doi: 10.3390/ijms21165640 (PMC7460654; doi:10.3390/ijms21165640)
Supplement: Supplementary file 1 [file ijms-21-05640-s001.pdf]

Supplementary Materials

Proteome-Wide Analyses Provide New Insights into the Compatible Interaction of Rice with Root-Knot Nematode *Meloidogyne graminicola*

Chao Xiang[1, †], Xiaoping Yang[2, †], Deliang Peng[1], Houxiang Kang[1], Maoyan Liu[1, 3], Wei Li[3], Wenkun Huang[1, *], Shiming Liu[1, *]

[1] State Key Laboratory for Biology of Plant Diseases and Insect Pests, Institute of Plant Protection, Chinese Academy of Agricultural Sciences, Beijing 100193, P. R. China

[2] Hunan Biological and Electromechanical Polytechnic, Changsha 410127, P. R. China

[3] College of Plant Protection, Hunan Agricultural University, Changsha 410128, P. R. China

[†] These authors equally contributed to this work

* Correspondence: smliuhn@yahoo.com (S.L.); wkhuang@ippcaas.cn (W.H.)

Figure S1. Volcano plots of the relative protein abundance changes of all the identified proteins in the roots of NPB with or without infection of *M. graminicola*.

Figure S2. Clustering heatmaps of the three biological replicates.

Figure S3. GO and KEGG enrichment analyses using all the specifically expressed proteins and SDEPs of NPB roots in CK1DPI_versus_CK0, CK3DPI_versus_CK0, or CK7DPI_versus_CK0.

Figure S4. GO and KEGG enrichment analyses of the specifically expressed proteins and SDEPs of the NPB roots in the whole CK_versus_CK0 group.

Figure S5. Compatible interaction of *japonica* rice cultivar Nipponbare (NPB) with *M. graminicola*.

Table S1 List of the specifically expressed proteins and significantly differentially expressed proteins (SDEPs) in the roots of rice NPB uniquely caused by the infection of *M. graminicola*.

Table S2 List of the primers used in this study.

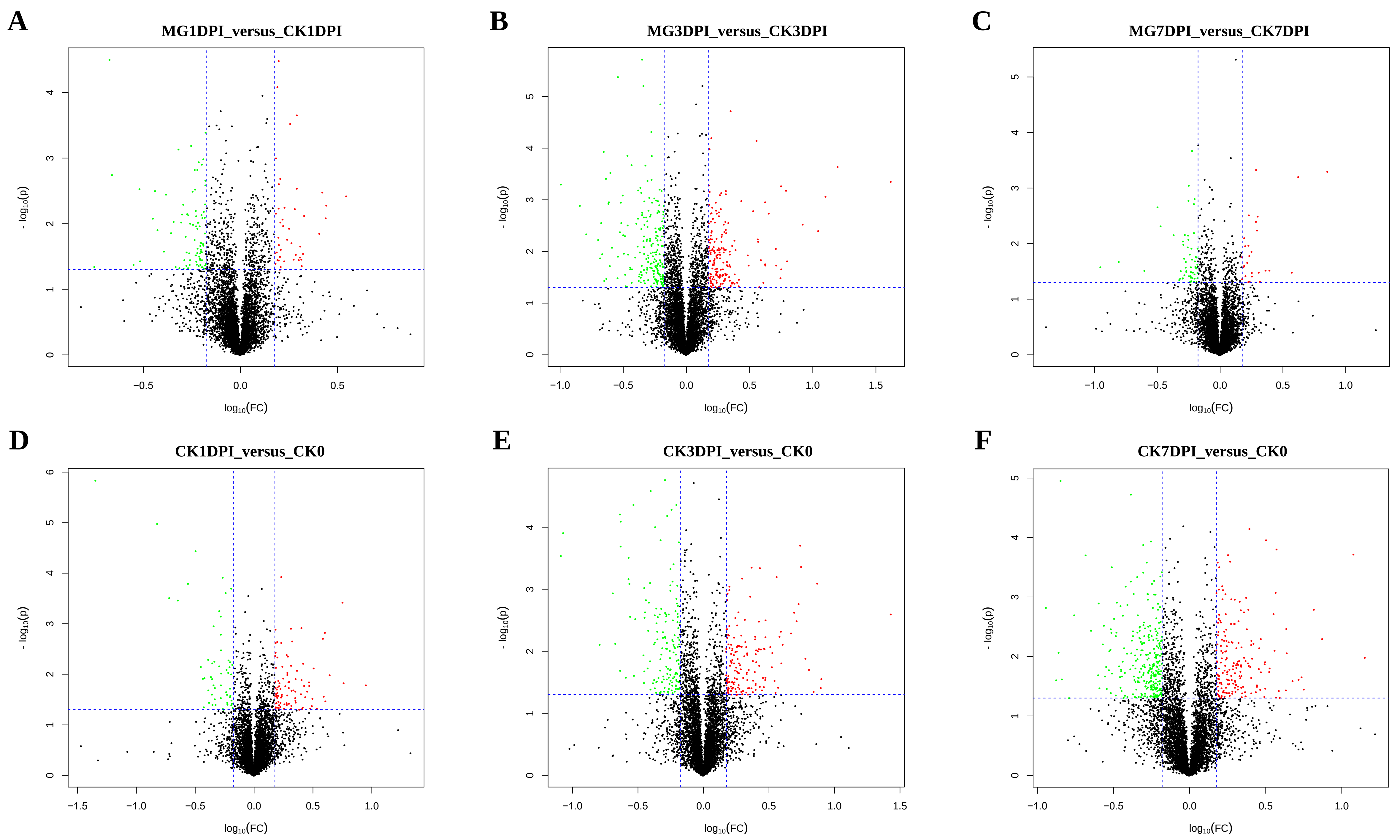

Figure S1. Volcano plots of the relative protein abundance changes of all the identified proteins in the roots of NPB with or without infection of *M. graminicola*. (**A**)-(**F**) Volcano plots of the relative protein abundance changes of all the identified proteins in MG1DPI_versus_CK1DPI, MG3DPI_versus_CK3DPI, MG7DPI_versus_CK7DPI, CK1DPI_versus_CK0, CK3DPI_versus_CK0, and CK7DPI_versus_CK0, respectively. Red and green dots indicate the proteins that are significantly up-regulated and significantly down-regulated, respectively.

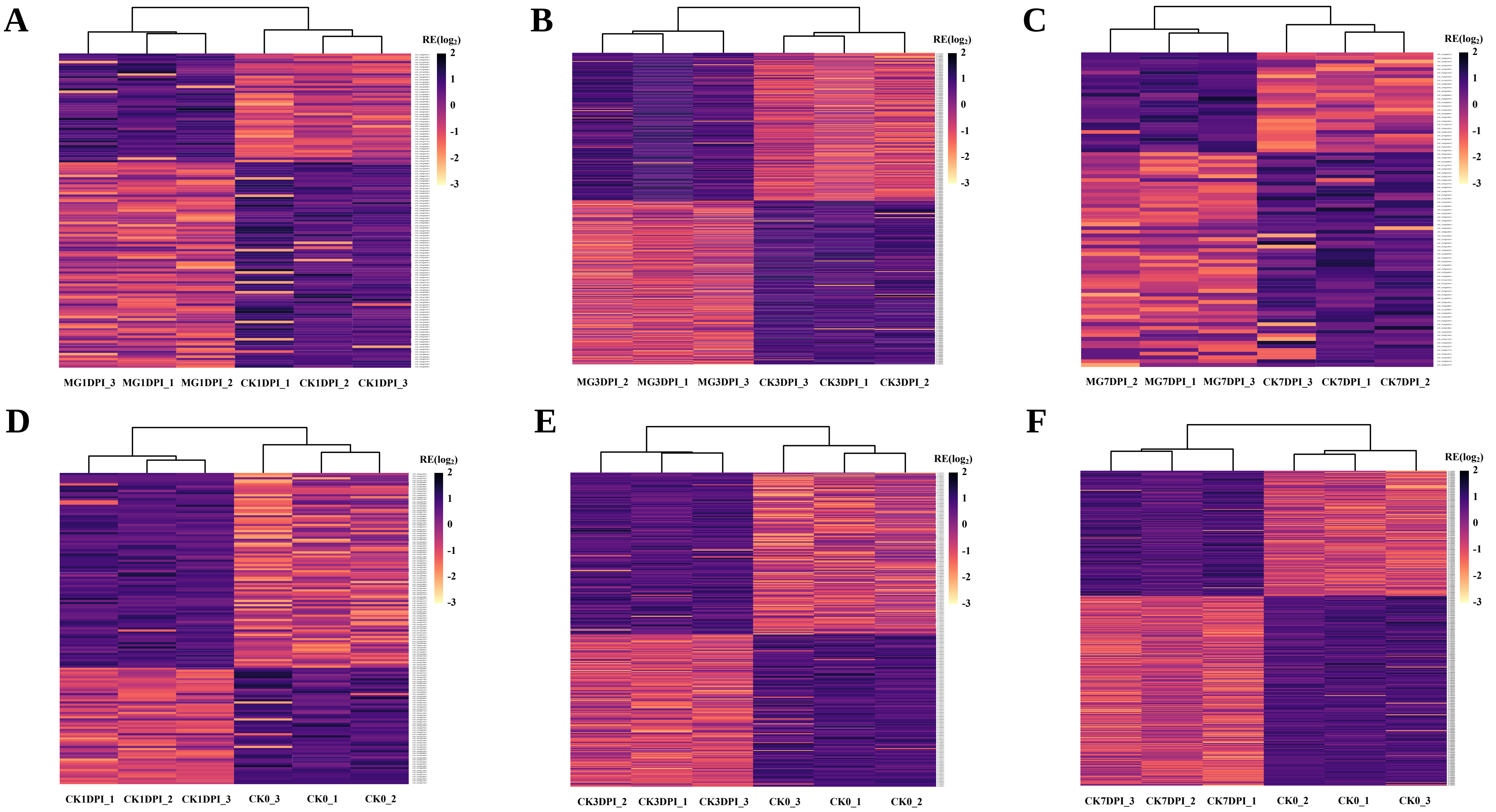

Figure S2. Clustering heatmaps of the three biological replicates. **(A)**-**(F)** Clustering heatmaps of MG1DPI_versus_CK1DPI, MG3DPI_versus_CK3DPI, MG7DPI_versus_CK7DPI, CK1DPI_versus_CK0, CK3DPI_versus_CK0, and CK7DPI_versus_CK0, respectively.

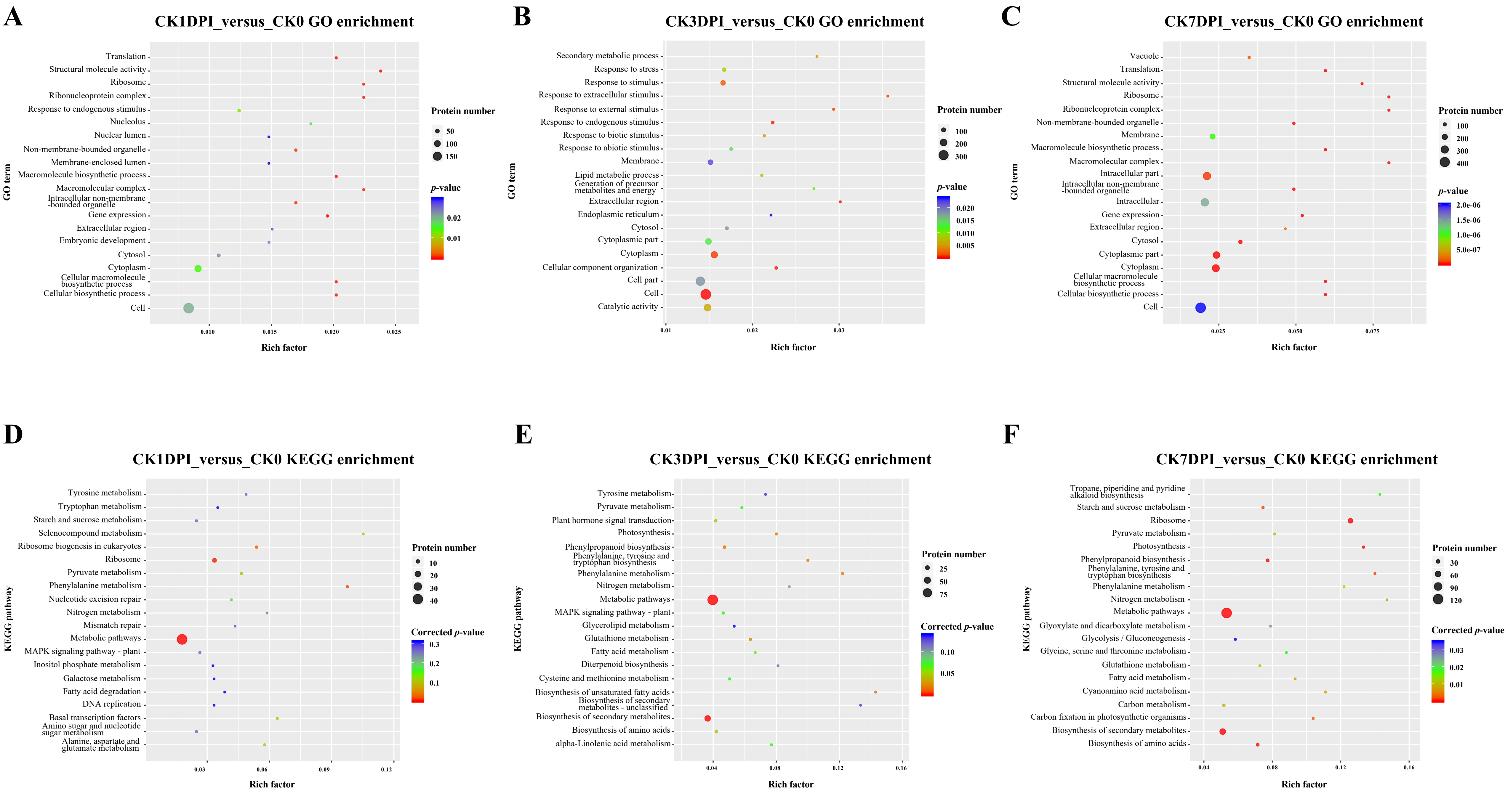

Figure S3. GO and KEGG enrichment analyses using all the specifically expressed proteins and SDEPs of NPB roots in CK1DPI_versus_CK0, CK3DPI_versus_CK0, or CK7DPI_versus_CK0. (**A**)-(**C**) GO enrichment analyses in CK1DPI_versus_CK0, CK3DPI_versus_CK0, and CK7DPI_versus_CK0, respectively. (**D**)-(**F**) KEGG enrichment analyses in CK1DPI_versus_CK0, CK3DPI_versus_CK0, and CK7DPI_versus_CK0, respectively.

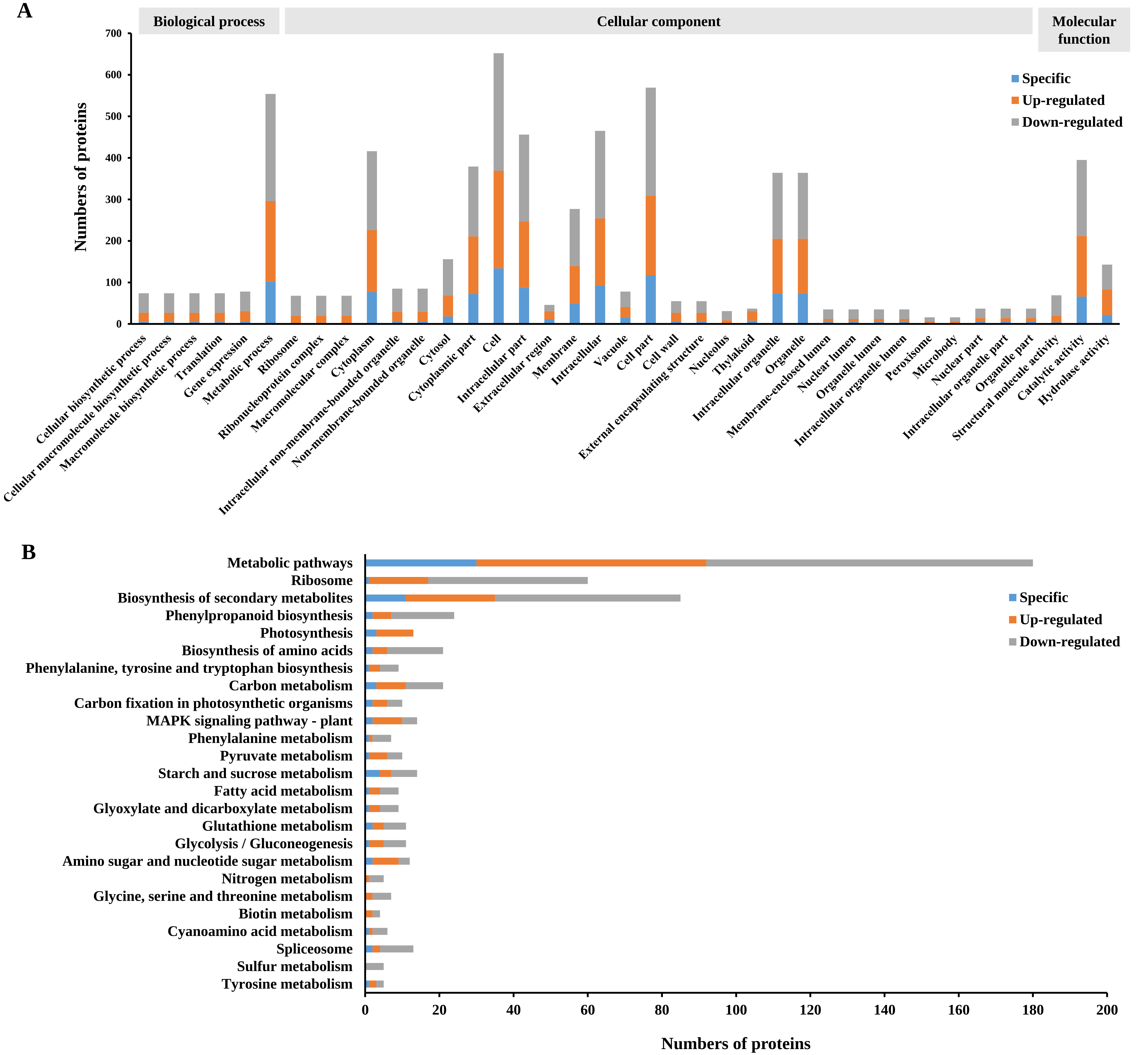

Figure S4. GO and KEGG enrichment analyses of all the specifically expressed proteins and SDEPs of the NPB roots in the whole CK_versus_CK0 group. (**A**) GO enrichment analyses. (**B**) KEGG pathway enrichment analyses.

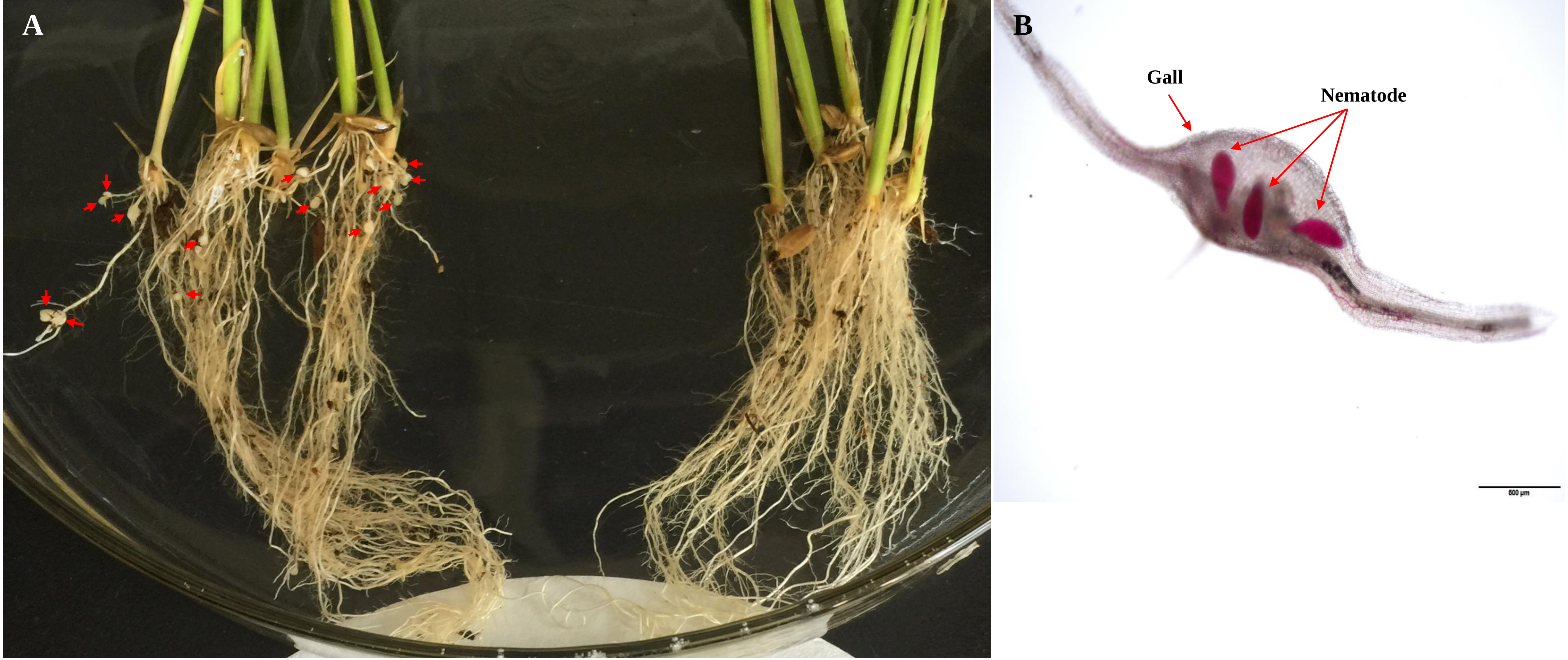

Figure S5. Compatible interaction of *japonica* rice cultivar Nipponbare (NPB) with *M. graminicola*. (**A**) Partial seedlings of NPB at 7 dpi of *M. graminicola*. Left and right pictures are the partial seedlings with and without inoculation, respectively. Many galls (red arrows directed) already emerged on the roots inoculated with *M. graminicola* at 7 dpi, but no galls appeared on the non-inoculated roots at the same growth day. (**B**) One representative gall on the roots with 3 stained nematodes (purple) inside observed under a stereomicroscopy at 7 dpi.

Table S1 List of the specifically expressed proteins and significantly differentially expressed proteins (SDEPs) in the roots of rice NPB uniquely caused by the infection of *M. graminicola* . Totally, 513 proteins were obtained. 'Specific', 'Up' and 'Down' denote specifically expressed proteins, up-regulated SDEPs and down-regulated SDEPs, respectively. The former 99 proteins were enriched on the KEGG pathways. The blue-highlighted are the speciallyselected proteins.

No.	Accession No.	Description	Gene ID	Expression	KEGG pathway
1	LOC_Os12g08780.1	flavin monooxygenase, putative, expressed	dosa:Os12t0189500-00	Specific	Tryptophan metabolism;Metabolic pathways
2	LOC_Os03g16210.1	tropinone reductase, putative, expressed	osa:4332375	Specific and Down	Tropane, piperidine and pyridine alkaloid biosynthesis;Metabolic pathways;Biosynthesis of secondary metabolites
3	LOC_Os11g25160.1	tropinone reductase 2, putative, expressed	dosa:Os11t0438700-01	Down	Tropane, piperidine and pyridine alkaloid biosynthesis;Metabolic pathways;Biosynthesis of secondary metabolites
4	LOC_Os12g09000.1	phosphomethylpyrimidine kinase/thiamin-phosphate pyrophosphorylase, putative, expressed	osa:4351707	Specific	Thiamine metabolism;Metabolic pathways
5	LOC_Os05g30454.1	thiamin pyrophosphokinase 1, putative, expressed	osa:4338566	Down	Thiamine metabolism;Metabolic pathways
6	LOC_Os03g52180.1	4-hydroxy-3-methylbut-2-enyl diphosphate reductase, putative, expressed	osa:4334004	Specific	Terpenoid backbone biosynthesis;Metabolic pathways;Biosynthesis of secondary metabolites
7	LOC_Os05g34180.1	hydrolase, NUDIX family, domain containing protein, expressed	osa:4338791	Up	Terpenoid backbone biosynthesis;Metabolic pathways;Biosynthesis of secondary metabolites
8	LOC_Os01g66360.1	2-C-methyl-D-erythritol 4-phosphate cytidylyltransferase, putative, expressed	osa:9266653	Down	Terpenoid backbone biosynthesis;Metabolic pathways;Biosynthesis of secondary metabolites
9	LOC_Os01g05810.1	gamma-glutamyltranspeptidase 1 precursor, putative, expressed	osa:4324525	Up	Taurine and hypotaurine metabolism;Cyanoamino acid metabolism;Glutathione metabolism;Arachidonic acid metabolism;Metabolic pathways
10	LOC_Os03g59040.2	squalene synthetase, putative, expressed	osa:4334492	Down	Steroid biosynthesis;Sesquiterpenoid and triterpenoid biosynthesis;Metabolic pathways;Biosynthesis of secondary metabolites
11	LOC_Os06g49970.2	alpha-amylase precursor, putative, expressed	osa:4342055	Up and Down	Starch and sucrose metabolism;Metabolic pathways
12	LOC_Os07g32600.1	glucan endo-1,3-beta-glucosidase precursor, putative, expressed	osa:4343350	Specific	Starch and sucrose metabolism;Metabolic pathways
13	LOC_Os02g47350.1	oxidoreductase, short chain dehydrogenase/reductase family, putative, expressed	osa:4330439	Up	Sphingolipid metabolism;Metabolic pathways
14	LOC_Os03g02600.1	riboflavin biosynthesis protein ribD, putative, expressed	dosa:Os03t0117200-01	Down	Riboflavin metabolism;Metabolic pathways;Biosynthesis of secondary metabolites

15	LOC_Os01g11054.1	phosphoenolpyruvate carboxylase, putative, expressed	osa:4325531	Down	Pyruvate metabolism;Carbon fixation in photosynthetic organisms;Metabolic pathways;Carbon metabolism
16	LOC_Os03g31750.3	pyruvate, phosphate dikinase, chloroplast precursor, putative, expressed	osa:4333181	Down	Pyruvate metabolism;Carbon fixation in photosynthetic organisms;Metabolic pathways;Carbon metabolism
17	LOC_Os01g70950.1	spermatogenesis-associated protein 20 precursor, putative, expressed	osa:4326794	Up	Pyrimidine metabolism;Metabolic pathways
18	LOC_Os06g02000.1	adenylate kinase, putative, expressed	osa:4339882	Down	Pyrimidine metabolism;Metabolic pathways
19	LOC_Os03g44660.1	cytosolic 5-nucleotidase III, putative, expressed	osa:4333582	Down	Purine metabolism;Pyrimidine metabolism;Nicotinate and nicotinamide metabolism;Metabolic pathways;Biosynthesis of secondary metabolites
20	LOC_Os05g08950.1	phosphoribosyl transferase, putative, expressed	osa:107276353	Up	Purine metabolism;Metabolic pathways;Biosynthesis of secondary metabolites
21	LOC_Os01g64520.1	uricase, putative, expressed	osa:4324793	Down	Purine metabolism;Metabolic pathways
22	LOC_Os05g06940.1	relA-SpoT like protein RSH4, putative, expressed	osa:9270381	Specific	Purine metabolism;Metabolic pathways
23	LOC_Os01g07770.1	peroxidase precursor, putative, expressed	osa:4327648	Up and Down	Phenylpropanoid biosynthesis;Metabolic pathways;Biosynthesis of secondary metabolites
24	LOC_Os12g08920.1	peroxidase precursor, putative, expressed	dosa:Os12t0191500-01	Up and Down	Phenylpropanoid biosynthesis;Metabolic pathways;Biosynthesis of secondary metabolites
25	LOC_Os11g40690.1	dehydrogenase, putative, expressed	dosa:Os11t0622800-00	Up and Down	Phenylpropanoid biosynthesis;Metabolic pathways;Biosynthesis of secondary metabolites
26	LOC_Os05g04500.1	peroxidase precursor, putative, expressed	osa:4337732	Up	Phenylpropanoid biosynthesis;Metabolic pathways;Biosynthesis of secondary metabolites
27	LOC_Os09g23530.1	dehydrogenase, putative, expressed	osa:4346988	Up	Phenylpropanoid biosynthesis;Metabolic pathways;Biosynthesis of secondary metabolites
28	LOC_Os03g25280.1	peroxidase precursor, putative, expressed	dosa:Os03t0368000-00	Down	Phenylpropanoid biosynthesis;Metabolic pathways;Biosynthesis of secondary metabolites
29	LOC_Os07g01410.1	peroxidase precursor, putative, expressed	sita:101755700	Down	Phenylpropanoid biosynthesis;Metabolic pathways;Biosynthesis of secondary metabolites
30	LOC_Os01g73200.1	peroxidase precursor, putative, expressed	osa:4324556	Down	Phenylpropanoid biosynthesis;Metabolic pathways;Biosynthesis of secondary metabolites
31	LOC_Os09g23560.1 (CAD)	dehydrogenase, putative, expressed	osa:4346993	Down	Phenylpropanoid biosynthesis;Metabolic pathways;Biosynthesis of secondary metabolites

32	LOC_Os02g14160.1	peroxidase precursor, putative, expressed	osa:4328832	Down	Phenylpropanoid biosynthesis;Metabolic pathways;Biosynthesis of secondary metabolites
33	LOC_Os03g22020.1	peroxidase precursor, putative, expressed	osa:9272450	Down	Phenylpropanoid biosynthesis;Metabolic pathways;Biosynthesis of secondary metabolites
34	LOC_Os03g25320.1	peroxidase precursor, putative, expressed	sita:101771235	Down	Phenylpropanoid biosynthesis;Metabolic pathways;Biosynthesis of secondary metabolites
35	LOC_Os06g06980.1	caffeoyl-CoA O-methyltransferase, putative, expressed	osa:4340240	Down	Phenylpropanoid biosynthesis;Flavonoid biosynthesis;Stilbenoid, diarylheptanoid and gingerol biosynthesis;Metabolic pathways;Biosynthesis of secondary metabolites;Biosynthesis of secondary metabolites - unclassified
36	LOC_Os04g33390.1	prephenate dehydratase domain containing protein, expressed	osa:4335756	Down	Phenylalanine, tyrosine and tryptophan biosynthesis;Metabolic pathways;Biosynthesis of secondary metabolites;Biosynthesis of amino acids
37	LOC_Os08g43370.1	6-phosphogluconolactonase, putative, expressed	osa:4346232	Down	Pentose phosphate pathway;Metabolic pathways;Biosynthesis of secondary metabolites;Carbon metabolism
38	LOC_Os07g08030.1	ribose-5-phosphate isomerase A, putative, expressed	osa:4342543	Up	Pentose phosphate pathway;Carbon fixation in photosynthetic organisms;Metabolic pathways;Biosynthesis of secondary metabolites;Carbon metabolism;Biosynthesis of amino acids
39	LOC_Os02g54880.1	cytochrome c oxidase subunit, putative, expressed	osa:4330974	Specific	Oxidative phosphorylation;Metabolic pathways
40	LOC_Os05g45730.1	ubiquinone oxidoreductase, putative, expressed	osa:4339427	Up	Oxidative phosphorylation;Metabolic pathways
41	LOC_Os07g31300.1	ATP synthase delta chain, mitochondrial precursor, putative, expressed	osa:4343288	Up	Oxidative phosphorylation;Metabolic pathways
42	LOC_Os08g01940.1	non-lysosomal glucosylceramidase, putative, expressed	dosa:Os08t0111200-01	Up and Down	Other glycan degradation;Sphingolipid metabolism;Metabolic pathways
43	LOC_Os03g60700.1	dolichyl-phosphate beta-glycosyltransferase, putative, expressed	osa:4334604	Up	N-Glycan biosynthesis;Metabolic pathways
44	LOC_Os11g47520.1	glycosyl hydrolase, putative, expressed	osa:4351187	Up	Metabolic pathways;Amino sugar and nucleotide sugar metabolism
45	LOC_Os05g15770.1	glycosyl hydrolase, putative, expressed	osa:4338208	Up	Metabolic pathways;Amino sugar and nucleotide sugar metabolism
46	LOC_Os11g47560.1	glycosyl hydrolase, putative, expressed	osa:4351191	Down	Metabolic pathways;Amino sugar and nucleotide sugar metabolism

47	LOC_Os01g33230.1	UDP-N-acetylglucosamine--N-acetylmuramyl-pyrophosphor yl-undecaprenol N-acetylglucosamine transferase, putative, expressed	dosa:Os01t0516700-01	Specific and Down	Metabolic pathways
48	LOC_Os02g54254.1	saccharopine dehydrogenase, putative, expressed	osa:4330940	Down	Lysine degradation;Metabolic pathways;Biosynthesis of secondary metabolites
49	LOC_Os03g09250.1	inositol-3-phosphate synthase, putative, expressed	osa:4331917	Down	Inositol phosphate metabolism;Metabolic pathways
50	LOC_Os02g33710.1	decarboxylase, putative, expressed	osa:4329593	Down	Histidine metabolism;Metabolic pathways;Biosynthesis of secondary metabolites
51	LOC_Os03g57220.1	hydroxyacid oxidase 1, putative, expressed	dosa:Os03t0786100-01	Up	Glyoxylate and dicarboxylate metabolism;Metabolic pathways;Biosynthesis of secondary metabolites;Carbon metabolism;Peroxisome
52	LOC_Os04g58710.1	AMP-binding domain containing protein, expressed	osa:4337447	Down	Glyoxylate and dicarboxylate metabolism;Metabolic pathways
53	LOC_Os01g67960.1	GPI transamidase component PIG-S-related, putative, expressed	osa:4324979	Up	Glycosylphosphatidylinositol (GPI)-anchor biosynthesis;Metabolic pathways
54	LOC_Os04g33190.1	AMP-binding enzyme, putative, expressed	osa:4335745	Down	Glycolysis / Gluconeogenesis;Pyruvate metabolism;Propanoate metabolism;Metabolic pathways;Biosynthesis of secondary metabolites;Carbon metabolism;Glyoxylate and dicarboxylate metabolism
55	LOC_Os02g51590.1	phosphoglycerate mutase, putative, expressed	osa:4330747	Up	Glycolysis / Gluconeogenesis;Glycine, serine and threonine metabolism;Metabolic pathways;Biosynthesis of secondary metabolites;Carbon metabolism;Biosynthesis of amino acids
56	LOC_Os04g38540.1	aldose 1-epimerase, putative, expressed	osa:4336039	Specific	Glycolysis / Gluconeogenesis;Galactose metabolism;Metabolic pathways;Biosynthesis of secondary metabolites
57	LOC_Os11g10480.1	dehydrogenase, putative, expressed	osa:4350053	Down	Glycolysis / Gluconeogenesis;Fatty acid degradation;Tyrosine metabolism;alpha-Linolenic acid metabolism;Metabolic pathways;Biosynthesis of secondary metabolites
58	LOC_Os04g20164.2	amine oxidase precursor, putative, expressed	dosa:Os04t0269600-01	Up	Glycine, serine and threonine metabolism;Tyrosine metabolism;Phenylalanine metabolism;beta-Alanine metabolism;Isoquinoline alkaloid biosynthesis;Tropane, piperidine and pyridine alkaloid biosynthesis;Metabolic pathways;Biosynthesis of secondary metabolites
59	LOC_Os09g26700.1	choline/ethanolamine kinase, putative, expressed	osa:4347160	Down	Glycerophospholipid metabolism;Metabolic pathways

60	LOC_Os03g27370.1	phospholipase D, putative, expressed	obr:102711051	Down	Glycerophospholipid metabolism;Ether lipid metabolism;Metabolic pathways;Biosynthesis of secondary metabolites;Endocytosis
61	LOC_Os03g50030.1	phospholipase A2, putative, expressed	osa:4333862	Specific	Glycerophospholipid metabolism;Ether lipid metabolism;Arachidonic acid metabolism;Linoleic acid metabolism;alpha-Linolenic acid metabolism;Metabolic pathways;Biosynthesis of secondary metabolites
62	LOC_Os01g49820.1	lipid phosphatase protein, putative, expressed	osa:4325649	Down	Glycerolipid metabolism;Glycerophospholipid metabolism;Biosynthesis of secondary metabolites
63	LOC_Os03g17480.1	IN2-1 protein, putative, expressed	osa:4332456	Up and Down	Glutathione metabolism;Metabolic pathways
64	LOC_Os07g28480.1	glutathione S-transferase, putative, expressed	osa:4343183	Up	Glutathione metabolism;Metabolic pathways
65	LOC_Os10g38670.1	glutathione S-transferase, putative, expressed	dosa:Os10t0530200-01	Up	Glutathione metabolism;Metabolic pathways
66	LOC_Os10g38489.1	glutathione S-transferase GSTU6, putative, expressed	dosa:Os10t0528400-01	Up	Glutathione metabolism;Metabolic pathways
67	LOC_Os10g38350.1	glutathione S-transferase, putative, expressed	osa:4349185	Up	Glutathione metabolism;Metabolic pathways
68	LOC_Os10g38730.1	glutathione S-transferase, putative, expressed	obr:102711734	Up	Glutathione metabolism;Metabolic pathways
69	LOC_Os03g39850.1 (GST)	glutathione S-transferase, putative, expressed	osa:4333371	Down	Glutathione metabolism;Metabolic pathways
70	LOC_Os04g56930.1	glycosyl hydrolases, putative, expressed	osa:4337313	Down	Galactose metabolism;Starch and sucrose metabolism;Metabolic pathways
71	LOC_Os05g51670.1	NAD dependent epimerase/dehydratase family protein, putative, expressed	osa:4339812	Down	Galactose metabolism;Amino sugar and nucleotide sugar metabolism;Metabolic pathways
72	LOC_Os06g03910.1	hydrolase, NUDIX family, domain containing protein, expressed	osa:4339996	Down	Fructose and mannose metabolism;Purine metabolism;Starch and sucrose metabolism;Metabolic pathways;Biosynthesis of secondary metabolites
73	LOC_Os04g20280.2	expressed protein	osa:4335343	Specific	Fatty acid elongation;Biosynthesis of unsaturated fatty acids;Biosynthesis of secondary metabolites;Fatty acid metabolism;Metabolic pathways
74	LOC_Os01g06600.1 (ACX)	glutaryl-CoA dehydrogenase, mitochondrial precursor, putative, expressed	osa:4324062	Down	Fatty acid degradation;alpha-Linolenic acid metabolism;Biosynthesis of unsaturated fatty acids;Metabolic pathways;Biosynthesis of secondary metabolites;Fatty acid metabolism;Peroxisome;Carbon metabolism;beta-Alanine metabolism;Propanoate metabolism
75	LOC_Os07g07450.1	versicolorin reductase, putative, expressed	osa:9270991	Up	Fatty acid biosynthesis;Biotin metabolism;Metabolic pathways;Fatty acid metabolism

76	LOC_Os04g09900.1	ent-kaurene synthase, chloroplast precursor, putative, expressed	osa:4335090	Up	Diterpenoid biosynthesis;Biosynthesis of secondary metabolites
77	LOC_Os01g22010.4	S-adenosylmethionine synthetase, putative, expressed	osa:4326996	Down	Cysteine and methionine metabolism;Metabolic pathways;Biosynthesis of secondary metabolites;Biosynthesis of amino acids
78	LOC_Os05g04510.1	S-adenosylmethionine synthetase, putative, expressed	osa:4337733	Down	Cysteine and methionine metabolism;Metabolic pathways;Biosynthesis of secondary metabolites;Biosynthesis of amino acids
79	LOC_Os12g41390.1	homocysteine S-methyltransferase protein, putative, expressed	osa:4352747	Up	Cysteine and methionine metabolism;Metabolic pathways;Biosynthesis of secondary metabolites
80	LOC_Os11g08380.1	1-aminocyclopropane-1-carboxylate oxidase, putative, expressed	osa:4349970	Up	Cysteine and methionine metabolism;Metabolic pathways;Biosynthesis of secondary metabolites
81	LOC_Os05g05680.1	1-aminocyclopropane-1-carboxylate oxidase, putative, expressed	osa:4337818	Down	Cysteine and methionine metabolism;Metabolic pathways;Biosynthesis of secondary metabolites
82	LOC_Os10g17650.1	Os10bglu34 - beta-glucosidase homologue, similar to Os3bglu6, expressed	osa:4348315	Down	Cyanoamino acid metabolism;Starch and sucrose metabolism;Phenylpropanoid biosynthesis;Metabolic pathways;Biosynthesis of secondary metabolites
83	LOC_Os11g33240.1	citrate synthase, putative, expressed	osa:4350660	Specific	Citrate cycle (TCA cycle);Glyoxylate and dicarboxylate metabolism;Metabolic pathways;Biosynthesis of secondary metabolites;Carbon metabolism;2-Oxocarboxylic acid metabolism;Biosynthesis of amino acids
84	LOC_Os02g47020.1	phosphoribulokinase/Uridine kinase family protein, expressed	osa:4330413	Specific	Carbon fixation in photosynthetic organisms;Metabolic pathways;Carbon metabolism
85	LOC_Os04g16680.1	fructose-1,6-bisphosphatase, putative, expressed	osa:4335227	Down	Carbon fixation in photosynthetic organisms;Metabolic pathways;Carbon metabolism
86	LOC_Os02g48560.1	fatty acid desaturase, putative, expressed	osa:4330523	Down	Biosynthesis of unsaturated fatty acids;Fatty acid metabolism;Metabolic pathways
87	LOC_Os09g23070.1	malonyl-CoA decarboxylase, mitochondrial precursor, putative, expressed	osa:4346967	Down	beta-Alanine metabolism;Propanoate metabolism;Metabolic pathways;Peroxisome
88	LOC_Os12g29760.1	oxidoreductase, aldo/keto reductase family protein, putative, expressed	osa:4352223	Down	Ascorbate and aldarate metabolism;Metabolic pathways;Biosynthesis of secondary metabolites
89	LOC_Os06g37080.1	L-ascorbate oxidase precursor, putative, expressed	osa:4341335	Down	Ascorbate and aldarate metabolism;Metabolic pathways

90	LOC_Os03g08530.1	aminotransferase, classes I and II, domain containing protein, expressed	dosa:Os03t0183600-00	Down	Arginine biosynthesis;Alanine, aspartate and glutamate metabolism;Carbon fixation in photosynthetic organisms;Metabolic pathways;Carbon metabolism;2-Oxocarboxylic acid metabolism;Biosynthesis of amino acids
91	LOC_Os04g01690.1	pyridoxal-dependent decarboxylase protein, putative, expressed	osa:4334917	Up	Arginine and proline metabolism;Metabolic pathways
92	LOC_Os07g04690.1	UDP-arabinose 4-epimerase 1, putative, expressed	osa:4342364	Down	Amino sugar and nucleotide sugar metabolism;Metabolic pathways
93	LOC_Os01g62020.1	NAD dependent epimerase/dehydratase family domain containing protein, expressed	osa:4327367	Down	Amino sugar and nucleotide sugar metabolism;Metabolic pathways
94	LOC_Os12g03470.1	alpha-N-arabinofuranosidase A, putative, expressed	osa:4351401	Down	Amino sugar and nucleotide sugar metabolism;Metabolic pathways
95	LOC_Os06g11240.1	12-oxophytodienoate reductase, putative, expressed	osa:4340486	Specific	alpha-Linolenic acid metabolism;Metabolic pathways;Biosynthesis of secondary metabolites
96	LOC_Os03g04000.1	AMP-binding domain containing protein, expressed	osa:4331515	Up	alpha-Linolenic acid metabolism;Metabolic pathways;Biosynthesis of secondary metabolites
97	LOC_Os02g12690.1	cytochrome P450, putative, expressed	osa:4328743	Up	alpha-Linolenic acid metabolism;Metabolic pathways;Biosynthesis of secondary metabolites
98	LOC_Os08g39300.1	aminotransferase, putative, expressed	osa:4345962	Down	Alanine, aspartate and glutamate metabolism;Glycine, serine and threonine metabolism;Glyoxylate and dicarboxylate metabolism;Metabolic pathways;Biosynthesis of secondary metabolites;Carbon metabolism;Peroxisome
99	LOC_Os08g36320.3	decarboxylase, putative, expressed	dosa:Os08t0465800-01	Up	Alanine, aspartate and glutamate metabolism;beta-Alanine metabolism;Taurine and hypotaurine metabolism;Butanoate metabolism;Metabolic pathways;Biosynthesis of secondary metabolites
100	LOC_Os08g35860.1	cytokinin dehydrogenase precursor, putative, expressed	osa:4345764	Down	Zeatin biosynthesis
101	LOC_Os03g57790.1	ubiquitin-conjugating enzyme, putative, expressed	osa:4334386	Down	Ubiquitin mediated proteolysis
102	LOC_Os03g04520.2	RNA recognition motif containing protein, putative, expressed	osa:4331556	Specific	Spliceosome
103	LOC_Os08g29650.1	RNA recognition motif containing protein, expressed	osa:4345455	Up	Spliceosome
104	LOC_Os07g47630.1	RNA recognition motif containing protein, expressed	dosa:Os07t0673500-01	Up	Spliceosome
105	LOC_Os10g06540.1 (VAMP)	vesicle-associated membrane protein, putative, expressed	osa:4348130	Down	SNARE interactions in vesicular transport
106	LOC_Os03g05020.1	PIR, putative, expressed	osa:4331593	Down	RNA transport
107	LOC_Os10g40040.1	expressed protein	osa:4349298	Specific	RNA degradation

108	LOC_Os11g43890.1	WD domain, G-beta repeat domain containing protein, expressed	osa:4351059	Up	RNA degradation
109	LOC_Os01g56190.1	HVT1, putative, expressed	dosa:Os01t0767700-01	Down	RNA degradation
110	LOC_Os10g42320.1	nonsense-mediated mRNA decay protein 3, putative, expressed	osa:4349477	Down	Ribosome biogenesis in eukaryotes;RNA transport
111	LOC_Os03g20100.1	S1 RNA binding domain containing protein, expressed	osa:4332652	Specific	Ribosome
112	LOC_Os02g10540.1	ribosomal protein L7/L12 C-terminal domain containing protein, expressed	osa:4328635	Up	Ribosome
113	LOC_Os03g40180.1	60S ribosomal protein L15, putative, expressed	osa:9266562	Down	Ribosome
114	LOC_Os01g54870.1	60S ribosomal protein L18a, putative, expressed	osa:4327004	Down	Ribosome
115	LOC_Os07g43310.1	ribosomal protein L7/L12 C-terminal domain containing protein, expressed	bdi:100837964	Down	Ribosome
116	LOC_Os07g14270.1	calreticulin precursor protein, putative, expressed	osa:4342826	Up	Protein processing in endoplasmic reticulum;Phagosome
117	LOC_Os04g36750.1	hsp20/alpha crystallin family protein, putative, expressed	osa:4335956	Down	Protein processing in endoplasmic reticulum
118	LOC_Os01g04330.1	OsCML16 - Calmodulin-related calcium sensor protein, expressed	osa:4325693	Up	Plant-pathogen interaction
119	LOC_Os02g46090.1 (CDPK)	CAMK_CAMK_like.15 - CAMK includes calcium/calmodulin depedent protein kinases, expressed	osa:4330351	Down	Plant-pathogen interaction
120	LOC_Os04g47300.1	CAMK_CAMK_like.26 - CAMK includes calcium/calmodulin depedent protein kinases, expressed	osa:4336653	Down	Plant-pathogen interaction
121	LOC_Os11g11960.1 (RPM1)	disease resistance protein RPM1, putative, expressed	osa:9267892	Down	Plant-pathogen interaction
122	LOC_Os09g39400.1	histidine-containing phosphotransfer protein, putative, expressed	osa:4347886	Specific	Plant hormone signal transduction
123	LOC_Os03g50860.1	histidine kinase, putative, expressed	osa:4333916	Specific	Plant hormone signal transduction
124	LOC_Os01g56200.1	BTBA2 - Bric-a-Brac,Tramtrack, Broad Complex BTB domain with Ankyrin repeat region, expressed	osa:4325503	Specific	Plant hormone signal transduction
125	LOC_Os11g05480.1	transcription factor, putative, expressed	osa:4349800	Up	Plant hormone signal transduction
126	LOC_Os01g59150.2	tubulin/FtsZ domain containing protein, putative, expressed	osa:4327550	Up	Phagosome
127	LOC_Os03g01530.1	tubulin/FtsZ domain containing protein, putative, expressed	osa:4331315	Up	Phagosome
128	LOC_Os01g18050.1	tubulin/FtsZ domain containing protein, putative, expressed	osa:4326917	Up	Phagosome
129	LOC_Os09g14510.1 (PEX3)	peroxin-3 family protein, putative, expressed	osa:4346691	Down	Peroxisome
130	LOC_Os05g02310.1	soluble inorganic pyrophosphatase, putative, expressed	osa:4337608	Up	Oxidative phosphorylation
131	LOC_Os04g48750.1	3-oxo-5-alpha-steroid 4-dehydrogenase, putative, expressed	osa:4336740	Down	N-Glycan biosynthesis
132	LOC_Os12g38380.1	tetratricopeptide repeat containing protein, putative, expressed	dosa:Os12t0571900-01	Specific	mRNA surveillance pathway
133	LOC_Os04g18010.2	cleavage and polyadenylation specificity factor subunit 1, putative, expressed	osa:9266269	Up	mRNA surveillance pathway
134	LOC_Os09g29980.1	transposon protein, putative, CACTA, En/Spm sub-class, expressed	osa:4347363	Up	mRNA surveillance pathway
135	LOC_Os08g21350.2	cig3, putative, expressed	osa:4345227	Down	mRNA surveillance pathway
136	LOC_Os07g14070.1	SCP-like extracellular protein, expressed	dosa:Os07t0243800-00	Up and Down	MAPK signaling pathway - plant;Plant hormone signal transduction;Plant-pathogen interaction

137	LOC_Os07g03730.1	SCP-like extracellular protein, expressed	osa:4342318	Down	MAPK signaling pathway - plant;Plant hormone signal transduction;Plant-pathogen interaction
138	LOC_Os03g49260.1	lipoxygenase, putative, expressed	osa:4333818	Down	Linoleic acid metabolism
139	LOC_Os03g40670.1	glycerophosphoryl diester phosphodiesterase family protein, putative, expressed	osa:4333407	Up	Glycerophospholipid metabolism
140	LOC_Os02g31030.2	glycerophosphoryl diester phosphodiesterase family protein, putative, expressed	osa:4329468	Up	Glycerophospholipid metabolism
141	LOC_Os05g15530.1 (AT)	aminotransferase domain containing protein, putative, expressed	osa:4338203	Down	Folate biosynthesis
142	LOC_Os03g25150.1	transposon protein, putative, unclassified, expressed	bdi:100842616	Up	Flavone and flavonol biosynthesis
143	LOC_Os09g32760.1	acyl-ACP thioesterase, putative, expressed	osa:4347505	Up	Fatty acid biosynthesis
144	LOC_Os04g51120.1	ENTH domain containing protein, expressed	osa:4336871	Down	Endocytosis
145	LOC_Os01g46580.1	actin-related protein 2/3 complex subunit 2, putative, expressed	osa:4324174	Down	Endocytosis
146	LOC_Os02g58640.1	tumor susceptibility gene 101, putative, expressed	osa:4331267	Down	Endocytosis
147	LOC_Os02g58220.1	RPA2A - Putative single-stranded DNA binding complex subunit 2, expressed	osa:4331236	Specific	DNA replication;Nucleotide excision repair;Mismatch repair;Homologous recombination
148	LOC_Os12g07720.1	RFC4 - Putative clamp loader of PCNA, replication factor C subunit 4, expressed	osa:4351654	Specific	DNA replication;Nucleotide excision repair;Mismatch repair
149	LOC_Os03g51030.1	phytochrome A, putative, expressed	osa:4333930	Down	Circadian rhythm - plant
150	LOC_Os01g58680.1	apurinic endonuclease-redox protein, putative, expressed	osa:4327960	Down	Base excision repair
151	LOC_Os04g42990.1	suppressor of stem-loop protein 1, putative, expressed	dosa:Os04t0508900-00	Specific	Basal transcription factors;Nucleotide excision repair
152	LOC_Os03g10070.1	transcription initiation factor IIB, putative, expressed	osa:4331943	Down	Basal transcription factors
153	LOC_Os08g09240.2	autophagy-related protein, putative, expressed	osa:4344857	Down	Autophagy - other
154	LOC_Os01g54020.1	tRNA synthetase, putative, expressed	osa:4326551	Up	Aminoacyl-tRNA biosynthesis
155	LOC_Os01g72740.1	cytochrome P450, putative, expressed	obr:102718882	Up	-
156	LOC_Os02g11030.1	50S ribosomal protein L19, chloroplast precursor, putative, expressed	obr:102713795	Specific	-
157	LOC_Os03g57450.1	CAMK_CAMK_like.2 - CAMK includes calcium/calmodulin depedent protein kinases, expressed	dosa:Os03t0788500-01	Up	-
158	LOC_Os06g08080.1	inorganic H+ pyrophosphatase, putative, expressed	osa:4340298	Up	-
159	LOC_Os05g11950.1	GDSL-like lipase/acylhydrolase, putative, expressed	dosa:Os05t0210100-01	Specific and Down	-
160	LOC_Os05g43100.1	GDSL-like lipase/acylhydrolase, putative, expressed	obr:102720987	Up and Down	-
161	LOC_Os03g25030.1	GDSL-like lipase/acylhydrolase, putative, expressed	dosa:Os03t0365800-01	Up and Down	-
162	LOC_Os05g43120.1	GDSL-like lipase/acylhydrolase, putative, expressed	obr:102720987	Up	-
163	LOC_Os01g12320.1	GDSL-like lipase/acylhydrolase, putative, expressed	sita:101772224	Up	-
164	LOC_Os12g16200.1	glutathione synthetase, chloroplast precursor, putative, expressed	obr:102718696	Up	-
165	LOC_Os01g08090.1	flavonol-3-O-glycoside-7-O-glucosyltransferase 1, putative, expressed	dosa:Os01t0176000-01	Up	-

166	LOC_Os02g09240.1	cytochrome P450 71D8, putative, expressed	dosa:Os02t0185200-01	Down	-
167	LOC_Os03g19350.1	OsLonP1 - Putative Lon protease homologue, expressed	osa:4332601	Specific and Down	-
168	LOC_Os02g52610.1	xyloglucan fucosyltransferase, putative, expressed	dosa:Os02t0763900-01	Specific and Down	-
169	LOC_Os03g01730.1	expressed protein	dosa:Os04t0119300-00	Specific	-
170	LOC_Os03g32526.1	tRNA-splicing endonuclease positive effector-related, putative, expressed	osa:4333212	Specific	-
171	LOC_Os05g23940.1	transcription initiation factor IIB, putative, expressed	osa:4338362	Specific	-
172	LOC_Os01g22390.2	WD domain containing protein, putative, expressed	osa:4326972	Specific	-
173	LOC_Os04g33450.1	expressed protein	osa:4335760	Specific	-
174	LOC_Os06g36490.1	ethylene-responsive element-binding protein, putative, expressed	osa:4341303	Specific	-
175	LOC_Os05g36070.1	signal peptide peptidase domain containing protein, expressed	osa:4338901	Specific	-
176	LOC_Os01g48900.1	dynamin-related protein 1A, putative, expressed	osa:9269791	Specific	-
177	LOC_Os07g35750.1	TKL_IRAK_DUF26-ld.3 - DUF26 kinases have homology to DUF26 containing loci, expressed	osa:4343505	Specific	-
178	LOC_Os07g46920.1	sex determination protein tasselseed-2, putative, expressed	osa:4344204	Specific	-
179	LOC_Os02g30620.2	dnaJ domain containing protein, expressed	osa:4329448	Specific	-
180	LOC_Os01g20160.1	OsHKT1;5 - Na+ transporter, expressed	osa:4327757	Specific	-
181	LOC_Os03g27820.1	expressed protein	osa:4333036	Specific	-
182	LOC_Os09g25720.1	glycine-rich cell wall structural protein 2 precursor, putative, expressed	dosa:Os09t0425500-01	Specific	-
183	LOC_Os04g45190.3	MSP domain containing protein, expressed	dosa:Os04t0534400-01	Specific	-
184	LOC_Os10g41560.1	expressed protein	osa:4349418	Specific	-
185	LOC_Os04g33240.1	sex determination protein tasselseed-2, putative, expressed	osa:4335749	Specific	-
186	LOC_Os04g21710.1	expressed protein	osa:4335381	Specific	-
187	LOC_Os03g58960.1	DHHC zinc finger domain containing protein, expressed	osa:4334485	Specific	-
188	LOC_Os08g16830.1	retrotransposon protein, putative, SINE subclass, expressed	osa:4345134	Specific	-
189	LOC_Os07g48410.1	RNA-binding zinc finger protein, putative, expressed	osa:4344312	Specific	-
190	LOC_Os09g04240.1	retrotransposon protein, putative, unclassified, expressed	pda:103699490	Specific	-
191	LOC_Os08g03440.1	actin, putative, expressed	osa:4344577	Specific	-
192	LOC_Os02g54500.1	WD40-like, putative, expressed	dosa:Os02t0786000-01	Specific	-
193	LOC_Os02g39140.1	helix-loop-helix DNA-binding domain containing protein, expressed	osa:4329910	Specific	-
194	LOC_Os12g10200.1	SAM-dependent methyltransferase, putative, expressed	dosa:Os12t0203100-01	Specific	-
195	LOC_Os03g21830.1	appr-1-p processing enzyme family protein, putative, expressed	osa:4332764	Specific	-
196	LOC_Os05g38270.1	regulator of chromosome condensation, putative, expressed	osa:107275700	Specific	-

197	LOC_Os03g43990.1	BTBN8 - Bric-a-Brac, Tramtrack, Broad Complex BTB domain with non-phototropic hypocotyl 3 NPH3 and coiled-coil domains, expressed	osa:107280491	Specific	-
198	LOC_Os01g71340.1	glycosyl hydrolases family 17, putative, expressed	osa:4326518	Specific	-
199	LOC_Os10g42870.1	peptide transporter PTR2, putative, expressed	osa:4349516	Specific	-
200	LOC_Os08g01920.1	SGR2, putative, expressed	osa:4344482	Specific	-
201	LOC_Os03g04130.1	AMP-binding domain containing protein, expressed	osa:4331525	Specific	-
202	LOC_Os04g50120.1	zinc-binding protein, putative, expressed	osa:4336825	Specific	-
203	LOC_Os09g02130.1	expressed protein	osa:4346382	Specific	-
204	LOC_Os01g57690.1	early nodulin 20 precursor, putative, expressed	osa:4327048	Specific	-
205	LOC_Os02g25700.1	O-acyltransferase, putative, expressed	osa:4329278	Specific	-
206	LOC_Os10g39640.1	expansin precursor, putative, expressed	osa:4349265	Specific	-
207	LOC_Os02g04450.1	plant-specific domain TIGR01589 family protein, putative, expressed	osa:4328235	Specific	-
208	LOC_Os02g13560.1	transporter family protein, putative, expressed	osa:4328798	Specific	-
209	LOC_Os08g03690.1	LTPL24 - Protease inhibitor/seed storage/LTP family protein precursor, expressed	osa:4344595	Specific	-
210	LOC_Os11g43600.1	peptide chain release factor protein, putative, expressed	osa:4351050	Specific	-
211	LOC_Os12g35630.1	elongation factor TS family protein, expressed	osa:4352441	Specific	-
212	LOC_Os07g13360.1	expressed protein	-	Specific	-
213	LOC_Os12g29560.1	DHHC zinc finger domain containing protein, expressed	osa:4352214	Specific	-
214	LOC_Os12g06210.1	harpin-induced protein 1 domain containing protein, expressed	osa:4351556	Specific	-
215	LOC_Os07g27810.1	fiber protein Fb34, putative, expressed	osa:4343166	Specific	-
216	LOC_Os08g43010.1	disease resistance RPP13-like protein 1, putative, expressed	osa:4346203	Specific	-
217	LOC_Os01g51640.1	expressed protein	osa:4325943	Specific	-
218	LOC_Os11g04460.1	calcium-transporting ATPase, plasma membrane-type, putative, expressed	osa:4349735	Specific	-
219	LOC_Os06g08280.1	protein kinase domain containing protein, expressed	osa:4340309	Specific	-
220	LOC_Os03g22140.1	tetratricopeptide repeat domain containing protein, expressed	osa:4332790	Specific	-
221	LOC_Os01g49120.1	MATE efflux family protein, putative, expressed	dosa:Os01t0684900-01	Specific	-
222	LOC_Os01g42234.2	amino acid permease family protein, putative, expressed	osa:4324220	Specific	-
223	LOC_Os05g05930.1	peripheral-type benzodiazepine receptor, putative, expressed	osa:9271175	Specific	-
224	LOC_Os08g41500.1	ubiquitin-associated protein, putative, expressed	osa:4346099	Specific	-
225	LOC_Os03g11010.1	natural resistance-associated macrophage protein, putative, expressed	osa:4332012	Specific	-
226	LOC_Os11g37940.1	WIP2 - Wound-induced protein precursor, expressed	osa:4350820	Specific	-
227	LOC_Os01g58620.1	expressed protein	osa:4327955	Specific	-
228	LOC_Os10g25870.1	dirigent, putative, expressed	osa:4348558	Specific	-

229	LOC_Os02g09780.1	phytoene dehydrogenase-like, putative, expressed	osa:4328575	Specific	-
230	LOC_Os02g58110.1	expressed protein	dosa:Os02t0827500-01	Specific	-
231	LOC_Os11g45990.1	von Willebrand factor type A domain containing protein, putative, expressed	osa:4351146	Specific	-
232	LOC_Os02g58030.1	leucine-rich repeat-containing protein 40, putative, expressed	osa:4331218	Specific	-
233	LOC_Os03g04380.1	LYR motif containing protein, putative, expressed	osa:4331544	Specific	-
234	LOC_Os05g01360.1	expressed protein	osa:4337547	Specific	-
235	LOC_Os08g01710.1	GLTP domain containing protein, putative, expressed	dosa:Os08t0108700-00	Specific	-
236	LOC_Os03g44670.1	expressed protein	osa:4333583	Specific	-
237	LOC_Os05g51630.2	early-responsive to dehydration protein-related, putative, expressed	osa:4339808	Specific	-
238	LOC_Os10g35490.1	hydrolase, alpha/beta fold family domain containing protein, expressed	dosa:Os10t0498000-01	Specific	-
239	LOC_Os01g33420.1	glycosyl hydrolase family protein 27, putative, expressed	osa:4323975	Specific	-
240	LOC_Os02g54980.1	pheophorbide a oxygenase, chloroplast precursor, putative, expressed	osa:4330982	Specific	-
241	LOC_Os02g58080.1	transporter, major facilitator family, putative, expressed	osa:4331221	Specific	-
242	LOC_Os08g28790.1	dirigent, putative, expressed	osa:4345418	Specific	-
243	LOC_Os06g23350.1	late embryogenesis abundant protein D-34, putative, expressed	osa:4340952	Specific	-
244	LOC_Os11g04020.1	major facilitator superfamily antiporter, putative, expressed	osa:4349704	Specific	-
245	LOC_Os03g61810.1	tRNA-specific adenosine deaminase, putative, expressed	osa:4334690	Specific	-
246	LOC_Os03g16850.1	dof zinc finger domain containing protein, putative, expressed	osa:107280245	Specific	-
247	LOC_Os12g36240.1	inhibitor I family protein, putative, expressed	dosa:Os12t0548700-01	Specific	-
248	LOC_Os01g37770.1	RWD domain containing protein, expressed	osa:4324673	Specific	-
249	LOC_Os03g57854.1	MBTB3 - Bric-a-Brac, Tramtrack, Broad Complex BTB domain with Meprin and TRAF Homology MATH domain, expressed	osa:4334388	Specific	-
250	LOC_Os11g11100.1	bZIP transcription factor domain containing protein, expressed	osa:4350088	Specific	-
251	LOC_Os01g49510.1	OsPOP3 - Putative Prolyl Oligopeptidase homologue, expressed	osa:4327946	Specific	-
252	LOC_Os05g39500.1	DUF640 domain containing protein, putative, expressed	osa:107278201	Up and Down	-
253	LOC_Os06g39040.1	dehydrogenase/reductase SDR family member 12, putative, expressed	osa:9267040	Up and Down	-
254	LOC_Os07g07860.1	LTPL76 - Protease inhibitor/seed storage/LTP family protein precursor, expressed	osa:4342528	Up and Down	-
255	LOC_Os07g17330.1	B12D protein, putative, expressed	osa:4342911	Up and Down	-
256	LOC_Os09g39090.1	vignain precursor, putative, expressed	osa:107276064	Up and Down	-
257	LOC_Os10g39360.1	aspartic proteinase nepenthesin precursor, putative, expressed	osa:107275604	Up and Down	-
258	LOC_Os10g39390.1	Eukaryotic aspartyl protease domain containing protein, expressed	osa:107279218	Up and Down	-

259	LOC_Os10g18760.1	dirigent, putative, expressed	osa:9270308	Up and Down	-
260	LOC_Os05g45460.1	POEI52 - Pollen Ole e I allergen and extensin family protein precursor, expressed	osa:4339414	Up and Down	-
261	LOC_Os04g28250.1	cysteine proteinase inhibitor precursor, putative, expressed	osa:4335551	Up and Down	-
262	LOC_Os08g13440.1	cupin domain containing protein, expressed	osa:4344999	Up and Down	-
263	LOC_Os03g26820.1	LTPL52 - Protease inhibitor/seed storage/LTP family protein precursor, expressed	osa:4332993	Up and Down	-
264	LOC_Os06g49100.1	retrotransposon protein, putative, unclassified, expressed	osa:4341996	Up and Down	-
265	LOC_Os09g26620.1	auxin-repressed protein, putative, expressed	osa:4347155	Up and Down	-
266	LOC_Os05g02820.1	protein transport protein-related, putative, expressed	osa:107281145	Up and Down	-
267	LOC_Os01g14590.1	pathogen-related protein, putative, expressed	osa:4327214	Up and Down	-
268	LOC_Os03g19290.1	mitochondrial import inner membrane translocase subunit Tim17, putative, expressed	osa:4332597	Up and Down	-
269	LOC_Os03g19427.1	nicotianamine synthase, putative, expressed	osa:4332608	Up and Down	-
270	LOC_Os05g18604.1	OsSCP28 - Putative Serine Carboxypeptidase homologue, expressed	osa:4338250	Up and Down	-
271	LOC_Os02g03120.1	endoglucanase, putative, expressed	dosa:Os02t0123700-01	Up and Down	-
272	LOC_Os09g34070.2	RNA recognition motif containing protein, expressed	osa:4347579	Up	-
273	LOC_Os02g15220.1	bromodomain containing protein, expressed	dosa:Os02t0250300-01	Up	-
274	LOC_Os01g53250.1	NADPH reductase, putative, expressed	osa:4324160	Up	-
275	LOC_Os11g34120.1	exportin 1, putative, expressed	osa:4350677	Up	-
276	LOC_Os08g16600.1	WD-40 repeat protein family, putative, expressed	osa:4345122	Up	-
277	LOC_Os04g09680.1	caffeic acid 3-O-methyltransferase, putative, expressed	dosa:Os04t0175900-01	Up	-
278	LOC_Os01g60650.1	non-green plastid inner envelope membrane protein, putative, expressed	osa:4327612	Up	-
279	LOC_Os03g44520.1	expressed protein	osa:4333573	Up	-
280	LOC_Os03g53360.1	transferase family protein, putative, expressed	osa:4334082	Up	-
281	LOC_Os09g16090.1	UDP-glucoronosyl/UDP-glucosyl transferase, putative, expressed	osa:4346756	Up	-
282	LOC_Os01g37825.1	M16 domain containing zinc peptidase, putative, expressed	osa:4324677	Up	-
283	LOC_Os11g26750.1	dehydrin, putative, expressed	osa:4350451	Up	-
284	LOC_Os05g51450.1	OsClp8 - Putative Clp protease homologue, expressed	osa:4339792	Up	-
285	LOC_Os10g29620.1	tyrosine protein kinase domain containing protein, putative, expressed	osa:4348697	Up	-
286	LOC_Os05g01500.1	tubulin-specific chaperone E, putative, expressed	osa:4337557	Up	-
287	LOC_Os09g38740.1	expressed protein	osa:4347849	Up	-
288	LOC_Os07g38300.1	ribosome recycling factor, putative, expressed	osa:4343662	Up	-

289	LOC_Os12g21700.1	zinc finger C-x8-C-x5-C-x3-H type family protein, expressed	osa:4352041	Up	-
290	LOC_Os12g26380.1	dirigent, putative, expressed	osa:107279766	Up	-
291	LOC_Os04g02150.1	tRNA methyltransferase, putative, expressed	osa:4334938	Up	-
292	LOC_Os10g18820.1	dirigent, putative, expressed	osa:4348341	Up	-
293	LOC_Os04g56430.1	cysteine-rich receptor-like protein kinase, putative, expressed	osa:4337274	Up	-
294	LOC_Os06g12990.1	tRNA methyltransferase, putative, expressed	osa:4340601	Up	-
295	LOC_Os07g06970.1	HEN1, putative, expressed	dosa:Os07t0164000-01	Up	-
296	LOC_Os02g18660.1	expressed protein	osa:4329046	Up	-
297	LOC_Os10g18870.1	dirigent, putative, expressed	osa:4348342	Up	-
298	LOC_Os07g07920.1	LTPL100 - Protease inhibitor/seed storage/LTP family protein precursor, expressed	osa:4342533	Up	-
299	LOC_Os07g07790.1	LTPL75 - Protease inhibitor/seed storage/LTP family protein precursor, expressed	osa:9270363	Up	-
300	LOC_Os11g26790.1	dehydrin, putative, expressed	osa:4350454	Up	-
301	LOC_Os08g26110.1	dirigent, putative, expressed	obr:102707330	Up	-
302	LOC_Os04g24340.1	phytase, putative, expressed	osa:4335443	Up	-
303	LOC_Os01g07890.1	expressed protein	dosa:Os01t0174100-01	Up	-
304	LOC_Os04g24328.1	jasmonate-induced protein, putative, expressed	osa:9267350	Up	-
305	LOC_Os03g46710.1	expressed protein	osa:4333683	Up	-
306	LOC_Os04g24319.1	jasmonate-induced protein, putative, expressed	osa:107276694	Up	-
307	LOC_Os06g22100.2	DNA binding protein, putative, expressed	osa:9271930	Up	-
308	LOC_Os05g37270.1	retrotransposon protein, putative, unclassified, expressed	sind:105171171	Up	-
309	LOC_Os01g62980.2	LTPL101 - Protease inhibitor/seed storage/LTP family protein precursor, expressed	dosa:Os01t0849000-02	Up	-
310	LOC_Os01g25030.1	dirigent, putative	osa:107278107	Up	-
311	LOC_Os06g08730.1	expressed protein	osa:4340345	Up	-
312	LOC_Os01g56820.1	AGAP000951-PA, putative, expressed	dosa:Os01t0775600-01	Up	-
313	LOC_Os04g40620.1	peptide methionine sulfoxide reductase, putative, expressed	osa:107277650	Up	-
314	LOC_Os06g22070.1	mitochondrial glycoprotein, putative, expressed	osa:4340910	Up	-
315	LOC_Os10g28030.1	OsPOP22 - Putative Prolyl Oligopeptidase homologue, expressed	osa:4348628	Up	-
316	LOC_Os08g14570.1	NADPH reductase, putative, expressed	osa:4345047	Up	-
317	LOC_Os02g44870.1	dehydrin, putative, expressed	osa:4330265	Up	-
318	LOC_Os04g25560.1	OsSCP23 - Putative Serine Carboxypeptidase homologue, expressed	osa:4335479	Up	-
319	LOC_Os07g27950.1	RING zinc finger protein, putative, expressed	osa:4343169	Up	-
320	LOC_Os03g59080.1	AMP-binding enzyme, putative, expressed	osa:4334496	Up	-
321	LOC_Os07g26110.1	membrane associated DUF588 domain containing protein, putative, expressed	osa:4343098	Up	-

322	LOC_Os03g38990.1	DNA-binding protein, putative, expressed	osa:4333346	Up	-
323	LOC_Os01g55700.1	NLI interacting factor-like phosphatase, putative, expressed	osa:4327028	Up	-
324	LOC_Os04g41950.1	calcium-binding mitochondrial protein anon-60Da, putative, expressed	osa:4336282	Up	-
325	LOC_Os03g05920.1	expressed protein	osa:4331660	Up	-
326	LOC_Os03g52040.2	OsSCP19 - Putative Serine Carboxypeptidase homologue, expressed	osa:9271773	Up	-
327	LOC_Os03g63930.1	acyl carrier protein, putative, expressed	osa:4334838	Up	-
328	LOC_Os03g12810.1	maf, putative, expressed	osa:9270576	Up	-
329	LOC_Os06g49770.1	LTPL86 - Protease inhibitor/seed storage/LTP family protein precursor, expressed	osa:4342041	Up	-
330	LOC_Os01g03680.1	BBTI8 - Bowman-Birk type bran trypsin inhibitor precursor, expressed	osa:4325989	Up	-
331	LOC_Os03g51150.1	expressed protein	osa:4333936	Up	-
332	LOC_Os02g51300.1	AP2 domain containing protein, expressed	osa:4330724	Up	-
333	LOC_Os03g01660.1	SKP1, putative, expressed	osa:4331327	Up	-
334	LOC_Os01g63210.1	SOUL heme-binding protein, putative, expressed	osa:4324804	Up	-
335	LOC_Os01g03360.1	BBTI5 - Bowman-Birk type bran trypsin inhibitor precursor, expressed	osa:9271230	Up	-
336	LOC_Os06g18140.1	UDP-glucoronosyl and UDP-glucosyl transferase domain containing protein, expressed	osa:4340787	Up	-
337	LOC_Os09g32840.1	nucleotide pyrophosphatase/phosphodiesterase, putative, expressed	osa:4347510	Up	-
338	LOC_Os07g47510.1	stress-related protein, putative, expressed	dosa:Os07t0671800-01	Up	-
339	LOC_Os08g14200.1	glycosyl hydrolases family 16, putative, expressed	osa:9271033	Up	-
340	LOC_Os01g03390.1	BBTI7 - Bowman-Birk type bran trypsin inhibitor precursor, expressed	osa:9267088	Up	-
341	LOC_Os01g48850.1	auxin-responsive protein, putative, expressed	osa:4324390	Up	-
342	LOC_Os06g02210.1	bark storage protein A precursor, putative, expressed	osa:4339897	Up	-
343	LOC_Os06g19640.1	39S ribosomal protein L46, mitochondrial precursor, putative, expressed	osa:4340823	Up	-
344	LOC_Os10g09850.1	EF hand family protein, putative, expressed	osa:4348196	Up	-
345	LOC_Os07g49110.1	D-alanine--D-alanine ligase family, putative, expressed	osa:4344370	Up	-
346	LOC_Os01g48680.2	two pore calcium channel protein 1, putative, expressed	osa:4325272	Up	-
347	LOC_Os05g08390.1	expressed protein	osa:9270309	Up	-
348	LOC_Os06g06000.1	expressed protein	osa:107277456	Up	-
349	LOC_Os04g26870.1	oxidoreductase, aldo/keto reductase family protein, putative, expressed	dosa:Os04t0337500-01	Up	-
350	LOC_Os07g06860.1	gibberellin receptor GID1L2, putative, expressed	osa:4342463	Up	-
351	LOC_Os09g28280.1	gibberellin receptor GID1L2, putative, expressed	osa:4347255	Up	-
352	LOC_Os08g41880.1	nucleotide pyrophosphatase/phosphodiesterase, putative, expressed	osa:4346129	Up	-

353	LOC_Os12g31780.1	nitrilase-associated protein, putative, expressed	osa:4352277	Up	-
354	LOC_Os03g63450.1	snRK1-interacting protein 1, putative, expressed	osa:4334803	Up	-
355	LOC_Os04g22080.1	retrotransposon protein, putative, unclassified, expressed	osa:4335390	Up	-
356	LOC_Os06g34790.1	DUF538 domain containing protein, putative, expressed	osa:4341223	Up	-
357	LOC_Os07g43070.1	expressed protein	osa:4343957	Up	-
358	LOC_Os10g35190.1	ZOS10-06 - C2H2 zinc finger protein, expressed	osa:4348994	Up	-
359	LOC_Os08g02060.1	expressed protein	osa:4344492	Up	-
360	LOC_Os08g31860.1	expressed protein	osa:4345562	Up	-
361	LOC_Os02g06930.1	protein kinase, putative, expressed	osa:4328408	Up	-
362	LOC_Os07g01560.1	transporter family protein, putative, expressed	osa:4342198	Up	-
363	LOC_Os01g18744.1	transferase family protein, putative, expressed	osa:4325160	Up	-
364	LOC_Os02g52290.1	peptidyl-prolyl cis-trans isomerase, FKBP-type, putative, expressed	osa:4330800	Up	-
365	LOC_Os11g01600.1	macrophage migration inhibitory factor, putative, expressed	osa:4351273	Up	-
366	LOC_Os09g28720.1	alpha/beta hydrolase fold, putative, expressed	osa:4347291	Up	-
367	LOC_Os07g29610.1	PQ loop repeat domain containing protein, expressed	osa:4343225	Up	-
368	LOC_Os02g43660.1	plastocyanin-like domain containing protein, putative, expressed	osa:4330178	Up	-
369	LOC_Os07g46910.1	sex determination protein tasselseed-2, putative, expressed	osa:4344203	Up	-
370	LOC_Os06g35650.1	reticuline oxidase-like protein precursor, putative, expressed	osa:4341259	Up	-
371	LOC_Os07g38620.1	pyridoxamine 5'-phosphate oxidase family protein, putative, expressed	osa:4343688	Up	-
372	LOC_Os02g28900.1	cytokinin-O-glucosyltransferase 2, putative, expressed	osa:4329386	Up	-
373	LOC_Os07g26630.1	aquaporin protein, putative, expressed	osa:4343119	Up	-
374	LOC_Os10g28120.1	glycosyl hydrolase, putative, expressed	osa:4348634	Up	-
375	LOC_Os12g40490.1	LIM domain-containing protein, putative, expressed	osa:4352699	Up	-
376	LOC_Os05g45110.1	anthocyanidin 5,3-O-glucosyltransferase, putative, expressed	osa:107276526	Up	-
377	LOC_Os08g03520.1	retrotransposon protein, putative, Ty1-copia subclass, expressed	osa:4344581	Up	-
378	LOC_Os10g37240.2	expressed protein	dosa:Os10t0516500-01	Up	-
379	LOC_Os03g46190.2	parafibromin, putative, expressed	osa:4333654	Up	-
380	LOC_Os01g74460.1	vacuolar fusion protein MON1 homolog A, putative, expressed	osa:4324304	Up	-
381	LOC_Os03g46770.1	RNA recognition motif containing protein, expressed	osa:4333690	Up	-
382	LOC_Os04g37690.1	RNA recognition motif containing protein, putative, expressed	dosa:Os04t0449900-01	Down	-
383	LOC_Os11g47970.1	AAA-type ATPase family protein, putative, expressed	osa:4351224	Down	-
384	LOC_Os09g36350.1	endoglucanase, putative, expressed	osa:4347685	Down	-
385	LOC_Os08g28190.1	actin, putative, expressed	osa:4345402	Down	-

386	LOC_Os05g02940.1	calcium-transporting ATPase 2, endoplasmic reticulum-type, putative, expressed	osa:4337647	Down	-
387	LOC_Os01g45830.1	sulfate transporter, putative, expressed	osa:4325919	Down	-
388	LOC_Os03g06980.1	nucleic acid binding protein, putative, expressed	dosa:Os03t0166000-01	Down	-
389	LOC_Os02g32690.1	pleiotropic drug resistance protein 15, putative, expressed	egu:105040086	Down	-
390	LOC_Os04g52540.1	retrotransposon protein, putative, unclassified, expressed	osa:4336991	Down	-
391	LOC_Os06g19620.1	SFT2, putative, expressed	osa:4340821	Down	-
392	LOC_Os01g70840.2	esterase, putative, expressed	osa:4326787	Down	-
393	LOC_Os10g26660.1	expressed protein	osa:4348588	Down	-
394	LOC_Os05g22990.1	expressed protein	osa:4338325	Down	-
395	LOC_Os06g38550.1	expressed protein	osa:4341393	Down	-
396	LOC_Os01g42030.1	mitochondrial chaperone BCS1, putative, expressed	osa:4324105	Down	-
397	LOC_Os05g04584.1	transferase family protein, putative, expressed	osa:4337739	Down	-
398	LOC_Os07g45950.1	expressed protein	dosa:Os07t0653000-00	Down	-
399	LOC_Os04g14654.1	Sec1 family transport protein, putative, expressed	dosa:Os04t0223000-01	Down	-
400	LOC_Os01g20206.1	methyltransferase, putative, expressed	osa:107278642	Down	-
401	LOC_Os01g41810.1	cytochrome P450 72A1, putative, expressed	osa:4324604	Down	-
402	LOC_Os02g18820.1	WD domain, G-beta repeat domain containing protein, expressed	osa:4329056	Down	-
403	LOC_Os05g38720.1	Lung seven transmembrane receptor domain containing protein, putative, expressed	osa:4339027	Down	-
404	LOC_Os11g25454.1	cytokinin-N-glucosyltransferase 1, putative, expressed	osa:4350423	Down	-
405	LOC_Os01g45110.1	anthocyanin 3-O-beta-glucosyltransferase, putative, expressed	osa:4326578	Down	-
406	LOC_Os09g37200.1	transferase family protein, putative, expressed	osa:4347748	Down	-
407	LOC_Os09g36300.1	OsLonP4 - Putative Lon protease homologue, expressed	osa:4347683	Down	-
408	LOC_Os01g65700.1	expressed protein	osa:4324836	Down	-
409	LOC_Os02g14929.1	T-complex protein, putative, expressed	osa:4328872	Down	-
410	LOC_Os05g02060.1	mitochondrial import inner membrane translocase subunit Tim17, putative, expressed	osa:4337595	Down	-
411	LOC_Os03g30430.1	nitrilase-associated protein, putative, expressed	osa:9269934	Down	-
412	LOC_Os04g42840.1	HEAT repeat family protein, putative, expressed	osa:4336347	Down	-
413	LOC_Os09g36860.1	acyl carrier protein, putative, expressed	osa:4347723	Down	-
414	LOC_Os02g01790.2	expressed protein	osa:4328027	Down	-
415	LOC_Os04g50890.1	secretory carrier-associated membrane protein, putative, expressed	osa:4336849	Down	-
416	LOC_Os01g50200.1	UDP-glucoronosyl and UDP-glucosyl transferase domain containing protein, expressed	osa:4325293	Down	-
417	LOC_Os09g28160.1	phosphate carrier protein, mitochondrial precursor, putative, expressed	osa:4347248	Down	-

418	LOC_Os06g49140.1	ribosomal RNA large subunit methyltransferase J, putative, expressed	osa:4342000	Down	-
419	LOC_Os01g12940.1	phosphorylase domain containing protein, putative, expressed	obr:102700482	Down	-
420	LOC_Os02g12760.1	integral membrane protein, putative, expressed	osa:9269252	Down	-
421	LOC_Os04g36660.1	calmodulin binding protein, putative, expressed	osa:4335948	Down	-
422	LOC_Os05g32130.1	expressed protein	obr:102712909	Down	-
423	LOC_Os05g19910.1	transferase family protein, putative, expressed	osa:4338283	Down	-
424	LOC_Os06g14490.1	calmodulin-binding heat-shock protein, putative, expressed	dosa:Os06t0256300-01	Down	-
425	LOC_Os02g38820.1	expressed protein	osa:4329888	Down	-
426	LOC_Os05g37230.1	retrotransposon protein, putative, Ty3-gypsy subclass, expressed	bdi:106866389	Down	-
427	LOC_Os11g06760.1	protein of unknown function domain containing protein, expressed	osa:4349880	Down	-
428	LOC_Os12g28137.1	ATPase 3, putative, expressed	osa:107275748	Down	-
429	LOC_Os07g36200.1	eukaryotic translation initiation factor-related, putative, expressed	osa:4343524	Down	-
430	LOC_Os10g30560.1	UDP-glucoronosyl and UDP-glucosyl transferase domain containing protein, expressed	dosa:Os10t0442400-01	Down	-
431	LOC_Os01g70200.1	exostosin family domain containing protein, expressed	osa:4327243	Down	-
432	LOC_Os05g07040.1	PHD finger protein, putative, expressed	osa:4337899	Down	-
433	LOC_Os02g45110.1	MT-A70 domain containing protein, expressed	osa:4330284	Down	-
434	LOC_Os01g09540.1	HAD superfamily phosphatase, putative, expressed	osa:4327297	Down	-
435	LOC_Os06g37640.1	expressed protein	osa:4341363	Down	-
436	LOC_Os03g62090.1	CESA5 - cellulose synthase, expressed	osa:4334710	Down	-
437	LOC_Os04g57020.1	expressed protein	osa:4337320	Down	-
438	LOC_Os03g45720.1	glyoxalase family protein, putative, expressed	osa:4333625	Down	-
439	LOC_Os08g02080.1	rickettsia 17 kDa surface antigen family protein, expressed	osa:4344494	Down	-
440	LOC_Os10g42940.1	miro, putative, expressed	osa:4349518	Down	-
441	LOC_Os04g40490.1	glycosyl hydrolase family 5 protein, putative, expressed	osa:4336181	Down	-
442	LOC_Os06g21550.1	thioredoxin domain-containing protein 17, putative, expressed	osa:4340888	Down	-
443	LOC_Os09g39960.1	dynamin family protein, putative, expressed	osa:4347929	Down	-
444	LOC_Os05g50490.1	X8 domain containing protein, expressed	osa:4339730	Down	-
445	LOC_Os11g02150.1	saposin-like type B, region 1 family protein, putative, expressed	osa:4349589	Down	-
446	LOC_Os06g38940.1	RMD5 homolog A, putative, expressed	osa:4341403	Down	-
447	LOC_Os04g59394.1	expressed protein	osa:4337495	Down	-
448	LOC_Os01g37000.1	carboxyl-terminal peptidase, putative, expressed	osa:4325841	Down	-
449	LOC_Os03g55110.1	26S proteasome non-ATPase regulatory subunit 10, putative, expressed	osa:4334173	Down	-
450	LOC_Os01g51570.1	glycosyl hydrolases family 17, putative, expressed	osa:4325938	Down	-

451	LOC_Os10g40710.1	expansin precursor, putative, expressed	osa:4349348	Down	-
452	LOC_Os05g09580.1	expressed protein	osa:4338014	Down	-
453	LOC_Os07g44940.1	ATP12 ATPase, putative, expressed	osa:4344085	Down	-
454	LOC_Os02g08490.1	chaperone protein clpB 1, putative, expressed	osa:4328515	Down	-
455	LOC_Os07g38580.1	zinc finger family protein, putative, expressed	osa:4343683	Down	-
456	LOC_Os10g32700.1	hypersensitive-induced response protein, putative, expressed	osa:4348831	Down	-
457	LOC_Os06g39140.1	hemoglobin-like protein HbO, putative, expressed	osa:4341418	Down	-
458	LOC_Os05g13970.2	phosphorylase domain containing protein, putative, expressed	osa:4338154	Down	-
459	LOC_Os03g42130.1	gibberellin 20 oxidase 2, putative, expressed	osa:4333459	Down	-
460	LOC_Os05g31140.1	glycosyl hydrolases family 17, putative, expressed	osa:4338611	Down	-
461	LOC_Os02g45480.1	nucleic acid binding protein, putative, expressed	osa:9271844	Down	-
462	LOC_Os03g58790.1	ATPase, putative, expressed	osa:4334467	Down	-
463	LOC_Os02g40500.1	OsGrx_C2.1 - glutaredoxin subgroup I, expressed	osa:4329999	Down	-
464	LOC_Os01g56420.1	ctr copper transporter family protein, putative, expressed	osa:4324610	Down	-
465	LOC_Os01g62260.1	thaumatin, putative, expressed	osa:4327387	Down	-
466	LOC_Os09g39070.1	thiol protease SEN102 precursor, putative, expressed	osa:4347868	Down	-
467	LOC_Os02g58250.1	expressed protein	osa:4331238	Down	-
468	LOC_Os10g36690.4	dehydration response related protein, putative, expressed	osa:4349089	Down	-
469	LOC_Os05g36010.1	OsSub47 - Putative Subtilisin homologue, expressed	osa:4338899	Down	-
470	LOC_Os06g16280.1	expressed protein	osa:4340740	Down	-
471	LOC_Os10g42630.1	expressed protein	osa:4349495	Down	-
472	LOC_Os02g33820.1	abscisic stress-ripening, putative, expressed	osa:4329601	Down	-
473	LOC_Os01g15340.1	flowering promoting factor-like 1, putative, expressed	osa:4326229	Down	-
474	LOC_Os02g57470.1	tetratricopeptide repeat containing protein, putative, expressed	osa:4331168	Down	-
475	LOC_Os04g24600.1	cysteine proteinase 1 precursor, putative, expressed	osa:4335455	Down	-
476	LOC_Os05g47700.1	LTPL152 - Protease inhibitor/seed storage/LTP family protein precursor, expressed	osa:4339526	Down	-
477	LOC_Os01g08470.1	retrotransposon protein, putative, unclassified, expressed	osa:4325349	Down	-
478	LOC_Os11g44430.1	protein kinase, putative, expressed	osa:4351079	Down	-
479	LOC_Os05g34500.1	expressed protein	osa:9268643	Down	-
480	LOC_Os05g01600.2	actin, putative, expressed	osa:4337566	Down	-
481	LOC_Os01g06460.1	uncharacterized Cys-rich domain containing protein, putative, expressed	osa:4324087	Down	-
482	LOC_Os03g56500.1	expressed protein	osa:4334292	Down	-
483	LOC_Os03g63700.1	AIG2-like family domain containing protein, expressed	osa:4334822	Down	-
484	LOC_Os06g19650.1	expressed protein	osa:4340824	Down	-

485	LOC_Os05g49840.1	phospholipase, putative, expressed	osa:4339680	Down	-
486	LOC_Os01g71090.1	xylanase inhibitor, putative, expressed	osa:4326530	Down	-
487	LOC_Os04g44900.1	lectin-like receptor kinase, putative, expressed	osa:4336489	Down	-
488	LOC_Os08g42590.3	mtN19, putative, expressed	osa:4346175	Down	-
489	LOC_Os07g29450.1	expressed protein	dosa:Os07t0477500-01	Down	-
490	LOC_Os11g01140.1	AGC_PVPK_like_kin82y.18 - ACG kinases include homologs to PKA, PKG and PKC, expressed	osa:4349531	Down	-
491	LOC_Os06g12180.1	jacalin-like lectin domain containing protein, expressed	osa:9269817	Down	-
492	LOC_Os07g41340.1	B12D protein, putative, expressed	osa:4343847	Down	-
493	LOC_Os03g25480.1	cytochrome P450, putative, expressed	osa:4332937	Down	-
494	LOC_Os02g38050.1	joka2, putative, expressed	osa:4329846	Down	-
495	LOC_Os02g40710.1	ammonium transporter protein, putative, expressed	osa:4330007	Down	-
496	LOC_Os01g71860.1	glycosyl hydrolases family 17, putative, expressed	osa:4325834	Down	-
497	LOC_Os04g40500.1	glycosyl hydrolase family 5 protein, putative, expressed	osa:107276528	Down	-
498	LOC_Os07g19530.1	peptidase, putative, expressed	osa:4342962	Down	-
499	LOC_Os02g40730.1	ammonium transporter protein, putative, expressed	osa:4330008	Down	-
500	LOC_Os03g15340.1	plastocyanin-like domain containing protein, putative, expressed	osa:4332309	Down	-
501	LOC_Os01g21070.1	endoglucanase, putative, expressed	osa:4327286	Down	-
502	LOC_Os03g54000.1	oligopeptide transporter, putative, expressed	osa:4334129	Down	-
503	LOC_Os04g44910.1	receptor like protein kinase, putative, expressed	osa:4336490	Down	-
504	LOC_Os11g39450.1	cysteine-rich receptor-like protein kinase 7 precursor, putative, expressed	dosa:Os11t0608300-01	Down	-
505	LOC_Os07g34900.1	aspartic proteinase nepenthesin precursor, putative, expressed	dosa:Os07t0533300-00	Down	-
506	LOC_Os12g38770.1	nucleotide pyrophosphatase/phosphodiesterase, putative, expressed	osa:9268839	Down	-
507	LOC_Os10g39380.1	aspartic proteinase nepenthesin-1 precursor, putative, expressed	osa:107276470	Down	-
508	LOC_Os04g33060.1	NAD dependent epimerase/dehydratase family protein, putative, expressed	osa:4335739	Down	-
509	LOC_Os03g16960.1	cysteine-rich repeat secretory protein 55 precursor, putative, expressed	osa:4332423	Down	-
510	LOC_Os01g70850.1	esterase, putative, expressed	osa:4326788	Down	-
511	LOC_Os07g48500.1	stress responsive protein, putative, expressed	dosa:Os07t0684000-01	Down	-
512	LOC_Os07g36130.1	core histone H2A/H2B/H3/H4, putative, expressed	osa:4343518	Down	-
513	LOC_Os06g12590.1	protein kinase, putative, expressed	osa:4340578	Down	-

Table S2 List of the primers used in this studay.

Primers	Forward	Reverse primer
LOC_Os11g11960.1	5'-ATCCCTAAGTTGGTGTCATCTC-3'	5'-TTTAGTTTGGGGAATGCCTTTG-3'
LOC_Os02g46090.1	5'-ATGGCAGTGGTTATATTACGGT-3'	5'-ATGCGACCATCATTATCCTGAT-3'
LOC_Os09g14510.1	5'-TATGCCTGAAGTGGAACTCTTT-3'	5'-TGTTCTAATGGCAAAATTCGGG-3'
LOC_Os01g06600.1	5'-GAATTGTTGGGTGGAAATGGAA-3'	5'-CGTATGTGCCTTCGTATGAGTA-3'
LOC_Os09g23560.1	5'-TCCTAGGCTCCTAGCTATGTAG-3'	5'-TTTCAAGTTTCCATCGTGTACG-3'
LOC_Os10g06540.1	5'-CCTCCACCATGCAAGACAGT-3'	5'-TCCACAGAAAGCAGCGATGA-3'
LOC_Os05g15530.1	5'-ATCTGCATCCAAAGCAAAGATC-3'	5'-TACTTGATCGAGCCATTCCTG-3'
LOC_Os03g39850.1	5'-GCTCAAGGGAGTAGCTTATGAG-3'	5'-CTTCTTGTGGATGGGGTTCTT-3'
LOC_Os01g22490.1	5'-ACCACTTCGACCGCCACTACT-3'	5'-ACGCCTAAGCCTGCTGGTT-3'
